# Two Distinct Mechanisms for Actin Capping Protein Regulation—Steric and Allosteric Inhibition

**DOI:** 10.1371/journal.pbio.1000416

**Published:** 2010-07-06

**Authors:** Shuichi Takeda, Shiho Minakata, Ryotaro Koike, Ichiro Kawahata, Akihiro Narita, Masashi Kitazawa, Motonori Ota, Tohru Yamakuni, Yuichiro Maéda, Yasushi Nitanai

**Affiliations:** 1 Structural Biology Research Center, Graduate School of Science, Nagoya University, Nagoya, Japan; 2 ERATO Actin Filament Dynamics Project, Japan Science and Technology Agency c/o RIKEN SPring-8 Center, Hyogo, Japan; 3 Graduate School of Information Science, Nagoya University, Nagoya, Japan; 4 Institute for Bioinformatics Research and Development, Japan Science and Technology Agency, Tokyo, Japan; 5 Department of Pharmacotherapy, Graduate School of Pharmaceutical Sciences, Tohoku University, Sendai, Japan; 6 Division of Biological Science, Graduate School of Science, Nagoya University, Nagoya, Japan; 7 Structural Biophysics Laboratory, RIKEN SPring-8 Center, Hyogo, Japan; Adolf-Butenandt-Institut, Germany

## Abstract

The actin capping protein (CP) tightly binds to the barbed end of actin filaments, thus playing a key role in actin-based lamellipodial dynamics. V-1 and CARMIL proteins directly bind to CP and inhibit the filament capping activity of CP. V-1 completely inhibits CP from interacting with the barbed end, whereas CARMIL proteins act on the barbed end-bound CP and facilitate its dissociation from the filament (called uncapping activity). Previous studies have revealed the striking functional differences between the two regulators. However, the molecular mechanisms describing how these proteins inhibit CP remains poorly understood. Here we present the crystal structures of CP complexed with V-1 and with peptides derived from the CP-binding motif of CARMIL proteins (CARMIL, CD2AP, and CKIP-1). V-1 directly interacts with the primary actin binding surface of CP, the C-terminal region of the α-subunit. Unexpectedly, the structures clearly revealed the conformational flexibility of CP, which can be attributed to a twisting movement between the two domains. CARMIL peptides in an extended conformation interact simultaneously with the two CP domains. In contrast to V-1, the peptides do not directly compete with the barbed end for the binding surface on CP. Biochemical assays revealed that the peptides suppress the interaction between CP and V-1, despite the two inhibitors not competing for the same binding site on CP. Furthermore, a computational analysis using the elastic network model indicates that the interaction of the peptides alters the intrinsic fluctuations of CP. Our results demonstrate that V-1 completely sequesters CP from the barbed end by simple steric hindrance. By contrast, CARMIL proteins allosterically inhibit CP, which appears to be a prerequisite for the uncapping activity. Our data suggest that CARMIL proteins down-regulate CP by affecting its conformational dynamics. This conceptually new mechanism of CP inhibition provides a structural basis for the regulation of the barbed end elongation in cells.

## Abstract 2

A crystallographic study reveals the structural basis for regulation by two different inhibitors of the actin capping protein, a critical factor controlling actin-driven cell motility.

## Author Summary

Actin is a ubiquitous eukaryotic protein that polymerizes into bidirectional filaments and plays essential roles in a variety of biological processes, including cell division, muscle contraction, neuronal development, and cell motility. The actin capping protein (CP) tightly binds to the fast-growing end of the filament (the barbed end) to block monomer association and dissociation at this end, thus acting as an important regulator of actin filament dynamics in cells. Using X-ray crystallography, we present the atomic structures of CP in complex with fragments of two inhibitory proteins, V-1 and CARMIL, to compare the modes of action of these two regulators. The structures demonstrate that V-1 directly blocks the actin-binding site of CP, thereby preventing filament capping, whereas CARMIL functions in a very different manner. Detailed comparison of several CP structures revealed that CP has two stable domains that are continuously twisting relative to each other. CARMIL peptides were found to bind across the two domains of CP on a surface distinct from its actin binding sites. We propose that CARMIL peptides attenuate the binding of CP to actin filaments by suppressing the twisting movement required for tight barbed end capping. Our comparative structural studies therefore have revealed substantial insights in the variety of mechanisms by which different actin regulatory factors function.

## Introduction

The actin capping protein (CP) specifically binds to the barbed end of actin filaments with a high affinity and prevents the addition and loss of the monomers at this dynamic end [1],[2]. CP is a heterodimeric protein composed of α- and β-subunits and the molecule displays a pseudo two-fold symmetry due to the resemblance of the tertiary structures between the two subunits [3]. CP caps the filament with its two independent actin binding sites at the C-terminus of each subunit (“tentacles”). The tentacles are functionally non-equivalent: the α-tentacle is more important than the β-tentacle and is responsible for the initial contact with the barbed end [4]. A recent cryo-electron microscopy (EM) study provided a structural model for the barbed end capping by CP [5]. The model depicted the α-tentacle, with its surrounding residues in the β-subunit, wedged between the two end actin protomers, which represents the primary contact between CP and actin. A mutational analysis revealed that three conserved basic residues in this region, CP (α) Lys256, Arg260, and Arg266 (in the chicken α1 isoform), are critical for the barbed end capping [5]. The β-tentacle was predicted to interact with a hydrophobic cleft on the surface of the terminal protomer to stabilize the capping [5].

A growing body of evidence indicates that CP is a key regulator of actin-based lamellipodial dynamics. In vitro, CP is one of the essential proteins required for the formation of the Arp2/3 complex-nucleated branched-actin arrays, which drive lamellipodial protrusion [6]. CP prevents the production of longer filaments and maintains the cytosolic G-actin pool to promote the Arp2/3 complex-based filament nucleation and branching [7]. In mammalian cells, CP depletion leads to the explosive formation of filopodia, rather than lamellipodia [8]. Thus, the local concentration of CP and its affinity to the barbed end are critical determinants of dendritic actin assembly. The dissociation of CP from the barbed end is a rare event (t_1/2_∼30 min) in actin polymerization assays using purified proteins. However, recent microscopic observations of cultured cells showed that the fluorescent speckle lifetime of CP bound to actin filament network structures is on the order of seconds [9],[10], suggesting that CP does not stably cap the barbed end in living cells.

At present, several molecules have been identified that affect the barbed end capping activity of CP. These regulators can be categorized in two groups: (1) indirect regulators that bind to actin filaments and protect the barbed end from CP and (2) direct regulators that bind CP and modulate its capping activity. Formin is an indirect regulator because it associates with the barbed end and allows filament elongation even in the presence of CP [11]. Ena/VASP is also assumed to antagonize the capping activity without interacting directly with CP [12]. Polyphosphoinositides, such as PIP_2_, bind directly to CP and reduce the capping activity in vitro [13],[14].

The V-1 and CARMIL proteins are the only direct CP regulatory proteins that have been reported. V-1, also known as myotrophin, is a 13 kDa ankyrin repeat protein that consists of four ankyrin repeat motifs; two full-repeats are sandwiched between additional incomplete motifs at each terminus [15]. V-1 has been implicated in a variety of cellular events, including catecholamine synthesis [16], cerebellar development [17], cardiac hypertrophy [18], and insulin secretion [19]. Although the precise functional roles of V-1 in these processes have not been clarified, it is possible that V-1 acts as a CP regulator in vivo, because V-1 was found to form a complex with CP in primary-cultured cells and cell lines in murine cerebella [20],[21].

CARMIL is a multi-domain protein that reportedly interacts with myosin I, Arp2/3 complex, and CP [22]. Down-regulation of CARMIL resulted in impaired motility in Dictyostelium and mammalian cells [22],[23]. Although CARMIL is a large protein (∼150 kDa), its CP interaction site has been narrowed down to a small region [23],[24], and a ∼20 amino acid sequence in this region [CP-binding motif; LXHXTXXRPK(6X)P] is shared with other proteins, CD2AP, CIN85, and CKIP-1 [25]. All of these proteins (CARMIL proteins) can interact with CP via this consensus motif [25]. CD2AP and its homologue CIN85 are adaptor proteins involved in various cellular processes, such as T-cell activation, apoptosis, and actin cytoskeleton dynamics [26]. CKIP-1 interacts with casein kinase 2 and recruits the enzyme to the plasma membrane [27].

Previous studies have demonstrated that the V-1 and CARMIL proteins inhibit CP in distinct manners. (1) V-1 bound to CP blocks actin filament capping, whereas the CP/CARMIL protein complex has lower barbed end capping activity (K_D_∼15 nM) than free CP (∼1 nM) [23],[28],[29]. (2) CARMIL acts on the barbed end-bound CP and facilitates its dissociation from the filament (called uncapping activity), but V-1 lacks this activity [23],[25],[28],[29]. (3) The two actin binding sites in CP, the α- and β-tentacles, are not involved in the CARMIL interaction, whereas V-1 recognizes these sites [23],[28]. (4) The CP binding fragment of CARMIL, including the CP-binding motif, has little secondary structure. In contrast, V-1 is a structured ankyrin repeat protein [15],[23].

Although previous studies have revealed the striking functional differences between the two direct CP regulators, the molecular mechanisms by which these proteins inhibit CP remain poorly understood. In particular, the mechanism by which the CARMIL proteins uncap the filament that is tightly bound by CP has remained enigmatic. In this study, we present the crystal structures of CP complexed with V-1 and with peptides derived from the CP-binding motif of CARMIL proteins. Together with biochemical and computational studies, we have elucidated two distinct mechanisms for CP regulation by V-1 and CARMIL proteins—steric hindrance and allosteric restriction of conformational fluctuations.

## Results

**Figure 1:**
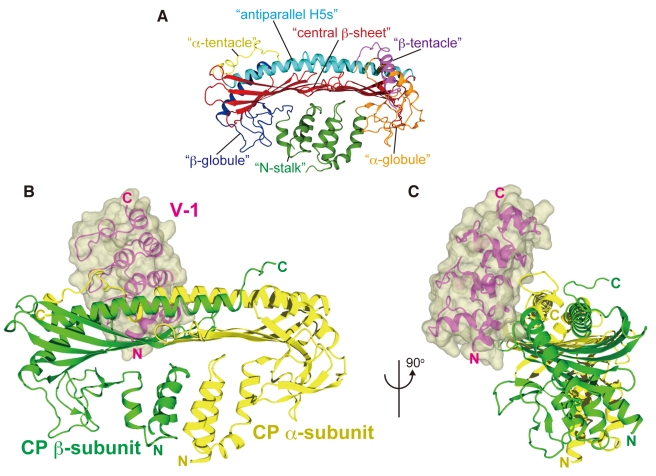
Crystal structure of the CP/V-1 complex. (A) The CP structural motifs are shown in different colors. (B and C) Two orthogonal views of the CP/V-1 complex structure are shown in ribbon models with the V-1 surface. The CP α-subunit, β-subunit, and V-1 are colored yellow, green, and magenta, respectively.

In this report, we describe the domain movement of CP. To facilitate the description, we refer to the structural motifs of CP as “N-stalk,” “α-globule,” “β-globule,” “central β-sheet,” “antiparallel H5s,” “α-tentacle,” and “β-tentacle” (Figure 1A; a detailed description of the motifs is provided in Figure S1).

### Crystal Structure of the CP/V-1 Complex

**Figure 2 pbio-1000416-g002:**
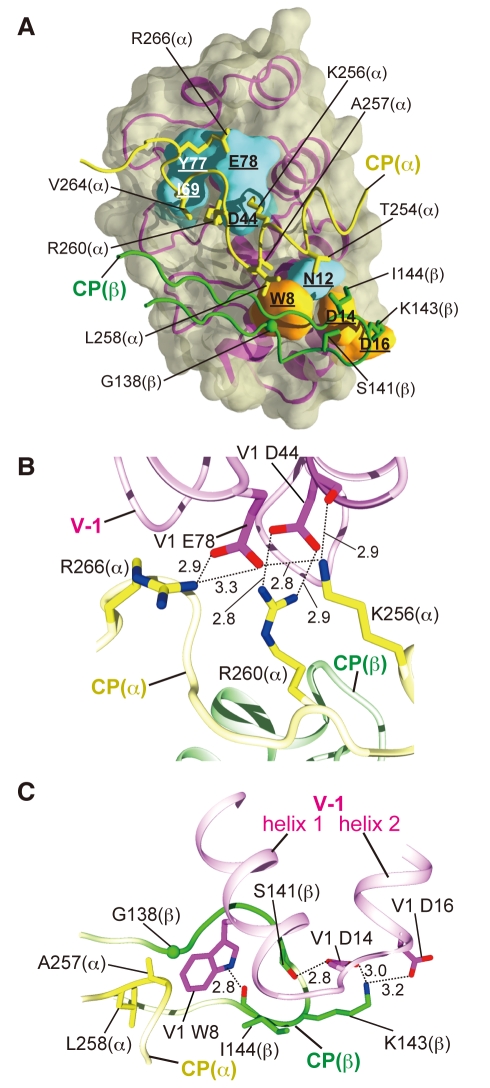
CP/V-1 molecular interface. (A) Residues involved in intermolecular interactions. V-1 residues contacting the CP α- or β-subunit residues are underlined and shown in cyan and orange, respectively. Interface CP residues are shown as stick models. (B) Interactions between the CP “basic triad” residues and V-1 Asp44 and Glu78. Salt bridges and hydrogen bonds are indicated by dotted lines with distances indicated in angstroms (B and C). (C) Interactions between V-1 Trp8 and the hydrophobic pocket adjacent to the CP “basic triad.”

To gain insight into the structural basis for the inhibition of CP by V-1, we solved the crystal structure of CP (chicken α1/β1) in complex with V-1 (human). The CP/V-1 complex was crystallized and the X-ray structure was determined at 2.2 Å resolution (*R* = 0.186, *R_free_* = 0.237) by molecular replacement, using the CP structure (PDB: 1IZN) as a search model (Figure 1B and 1C, and Table S1). CP contacts V-1 at two binding sites: (1) the basic residues at the C-terminus of the α-subunit and (2) a hydrophobic pocket adjacent to the basic contact site described above (Figures 2A and S1).

Three conserved basic residues in the CP α-subunit, Lys256, Arg260, and Arg266, were shown to be critical for the barbed end capping [5]. Remarkably, this “basic triad” directly participates in the V-1 interaction (Figure 2B). Arg260, the center of the “basic triad,” forms a bidentate salt bridge with V-1 Asp44. In addition, Lys256 and Arg266 form salt bridges with V-1 Glu78. Furthermore, Lys256 also forms a hydrogen bond with the main chain oxygen of V-1 Asp44. These notable ion pairs involving the “basic triad” clearly indicate that V-1 specifically binds conserved residues important for the interaction with actin, thereby effectively abolishing the barbed end capping. The importance of these ion pairs for complex formation was confirmed by a mutational analysis. We determined the CP/V-1 binding affinity by surface plasmon resonance measurements. Mutations of residues which form the “basic triad,” or their ion-pairing residues in V-1, reduced the affinity more than 25-fold compared with the wild type proteins (K_D_ = 21 nM: binding constants for the mutant proteins are summarized in Table S2). The effects of mutations in the “basic triad” on the V-1 interaction are similar to those on the barbed end capping: reverse-charged mutants have lower affinities for V-1 than alanine mutants, and multiple mutations exhibit more severe defects than single mutations [5].

Another striking feature in the CP/V-1 interface is the hydrophobic contact formed around V-1 Trp8 (Figure 2C). In V-1, Trp8 on the V-1 helix 1 inserts its indole ring into a hydrophobic pocket, which is formed by CP (α) Ala257 and Leu258, immediately adjacent to the “basic triad,” and CP (β) Gly138 and Ile144 in “loop S5–S6” (a loop connecting β-strands 5 and 6 of the β-subunit). This hydrophobic contact is further stabilized by a hydrogen bond between the aromatic nitrogen of the tryptophan and the main chain oxygen of CP (β) Ile144. Mutation of this tryptophan, V-1 W8A, drastically reduced the affinity for CP (K_D_ = 6.4 µM).

As expected, the CP binding-deficient V-1 did not inhibit CP in an actin polymerization assay (Figure S2). The wild-type V-1 allowed actin elongation from spectrin-actin seeds, even in the presence of CP. In contrast, the CP-binding deficient V-1 mutants (V-1 W8A, D44R, or E78R) had little inhibitory effect on CP activity.

### V-1 Sterically Hinders CP from Capping the Barbed End

**Figure 3:**
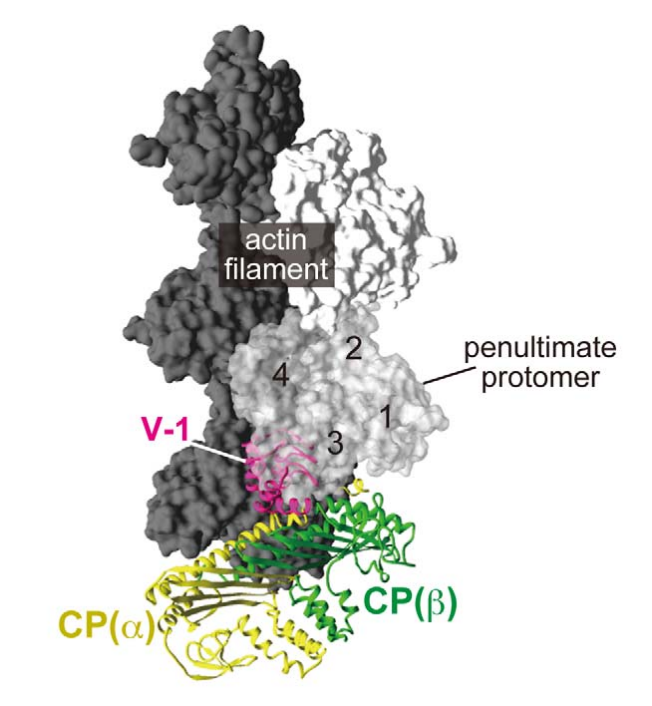
V-1 sterically hinders CP from the barbed end. Superposition of the CP/V-1 complex onto the EM model of the CP/barbed end structure. The CP/V-1 complex was superposed over the Cα positions of CP (β) 47–173 in the original CP model. The actin proto-filament is shown in a surface model (white or gray). The penultimate protomer (subdomains 1–4 are labeled) is transparent to show the steric hindrance by V-1.

We superposed the structure of the CP/V-1 complex onto the previous EM model of CP on the barbed end of an actin filament (Figure 3) [5]. This unambiguously demonstrated the collision of a major part of the V-1 molecule with the filament, mainly with subdomain 3 of the penultimate protomer. Furthermore, V-1 should prevent CP from even an initial contact with the barbed end, as it masks the “α-tentacle” by interacting with the “basic triad” residues (Figure 2B). Collectively, V-1 completely inhibits CP from interaction with the actin filament. The structure also indicates that V-1 lacks uncapping activity, because the V-1 binding site on CP is buried deeply between the two end protomers when CP caps the filaments.

### V-1 Overexpression Enhances Actin Polymerization in PC12D Cells

**Figure 4:**
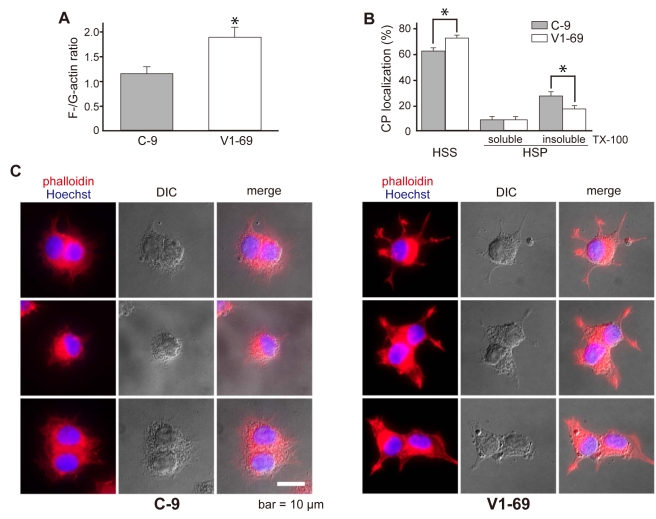
Overexpression of V-1 enhances actin polymerization in PC12D cells. (A) F-/G-actin ratio. The amounts of cellular actin in high-speed supernatants (G-actin) and pellets (F-actin) were quantified by Western blotting using an anti-actin antibody. Values are means ± SEM (*n* = 3). Statistical analysis was performed using Student's *t*-test (*p<0.05). (B) Subcellular fractionation of CP. “high speed supernatant (HSS),” “high speed pellet (HSP) soluble in detergent,” and “high speed pellet insoluble in detergent” fractions were prepared (see Materials and Methods) and subjected to a Western blot analysis, using an anti-CP β antibody (*n* = 3). Values are means ± SEM (*n* = 3). For statistical analysis, one-way ANOVA, followed by post hoc correction according to Tukey, was performed (*p<0.05). (C) Cell morphology. Alexa Fluor 546-conjugated phalloidin (red) and Hoechst 33258 (blue) fluorescence (left), DIC (middle), and the merged (right) images of C-9 and V1-69 are shown. Scale bar = 10 µm.

Although the association of V-1 with CP has been reported in vivo [20],[21], it remains unknown whether V-1 is involved in the regulation of cellular actin assembly. We addressed this question by using the rat neuronal PC12D cell line V1-69, which is stably transfected with V-1 cDNA and expresses a 5- to 6-fold higher amount of V-1 than the mock transfectant C-9 [16]. Initially, we measured the ratio of F-actin to G-actin by a sedimentation assay and found that more actin pelleted from extracts of V1-69 cells than mock cells (Figure 4A). This indicates that the overexpression of V-1 leads to enhanced actin polymerization in PC12D cells. We next examined the amount of CP in subcellular fractions. In the V1-69 cells, the proportion of CP in the “high speed supernatant” fraction was significantly larger than that of the mock transfectant. This result was inversely correlated with a decrease in the distribution of the “high speed pellet insoluble in detergent” fraction (Figure 4B: see Materials and Methods for the subcellular fractionation procedure). The overexpression of V-1 did not alter the total amount of CP in the transfectants (unpublished data). These results imply that V-1 enhances actin polymerization by inhibiting the interaction of CP with the cytoskeleton structures. Moreover, we observed that, compared to the mock cells, V1-69 cells exhibited membrane protrusive structures with a thick, neurite-like appearance (Figure 4C). Phalloidin staining revealed that these protrusions were enriched with actin filaments (Figure 4C), implying that CP suppression caused by V-1 overexpression leads to the alteration of cell morphology presumably due to the increase in the level of actin polymerization. Taken together, our results demonstrate the possible involvement of V-1 in the regulation of actin polymerization and cellular morphology in living cells.

### CP Consists of Two Rigid Domains and Undergoes Conformational Changes

**Figure 5:**
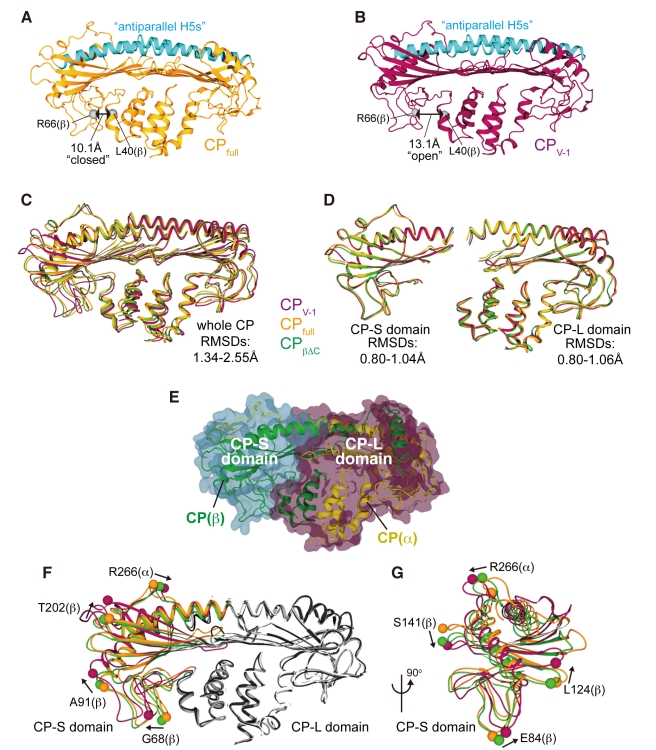
CP consists of two rigid domains and undergoes conformational changes. (A and B) Ribbon presentations of CP_full_ (orange) and CP_V-1_ (purple). The “antiparallel H5s” are highlighted in cyan and the Cα atoms of CP (β) Leu40 and Arg66 are represented as gray balls. (C) Superposition of CP_full_ (orange), CP_βΔC_ (green), and CP_V-1_ (purple) over the Cα positions of the entire CP molecule [residues 9–275 (α) and 3–244 (β)}. (D) Superposition of the CP-L and CP-S domains. (E) Surface presentation of the CP-L (purple) and CP-S (cyan) domains. Note that the domain boundary does not correspond to the subunit interface. (F and G) Twisting of CP-S relative to CP-L. CP_full_ (CP-L is shown in white), CP_βΔC_ (CP-L; gray), and CP_V-1_ (CP-L; black) are superimposed over CP (α) 9–257 in CP-L. Two orthogonal views are shown. To facilitate comparison, some residues are indicated as balls. In (G), CP-L was removed for clarity.

With the exception of the mobile “β-tentacle,” CP has been considered to be a rigid heterodimeric protein that is stabilized by many intra- and inter-subunit interactions [3]. However, we found that the overall conformation of V-1-bound CP (CP_V-1_; Figure 5B) is apparently different from the free form (CP_full_; PDB; 1IZN; Figure 5A); e.g., the “antiparallel H5s” is straighter and the “N-stalk” and “β-globule” are further apart. Superposition of the two structures was poor, with a root-mean-square displacement (RMSD) over the Cα atoms of 2.55 Å [residues 9–275 (α) and 3–244 (β); the “β-tentacle” was not included] (Figure 5C). This unexpected finding indicates that CP has conformational flexibility. For further structural comparison, we obtained a new ligand-free CP structure crystallized under different conditions from 1IZN (CP_βΔC_; at a 1.9 Å resolution) (Figure S3) and found that the structure of CP_βΔC_ is substantially different from both CP_full_ and CP_V-1_ (RMSDs of 1.34 Å and 1.87 Å, respectively) (Figure 5C and Table S3). These values are much larger than those expected for the same protein crystallized under different conditions (∼0.8 Å) [30]. Therefore, we conclude that CP conformational changes are not induced solely by the binding of a ligand molecule but show that CP is an intrinsically flexible molecule.

A domain motion analysis revealed that CP comprises two structurally stable domains, and the conformational change can be attributed to a twisting movement between the domains (Figures 5D–G and S4). The larger domain contains roughly two-thirds of the CP residues [residues 1–258 (α): 1–42, 175–192, and 235–277 (β)] and consists of the entire “N-stalk,” “α-globule,” and “β-tentacle” motifs together with parts of the “central β-sheet” and “antiparallel H5s,” whereas the smaller domain [residues 259–286 (α): 43–174 and 193–234 (β)] consists of the remaining portion. We refer to these larger and smaller domains as the CP-L and CP-S domains, respectively. Each domain superimposed well across the three forms (RMSDs of 0.80–1.06 Å for the CP-L domain and 0.80–1.04 Å for the CP-S domain) (Table S3). The boundary of the two domains does not directly correspond to the subunit interface; it resides between the “N-stalk” and “β-globule.” The two domains are linked by flexible regions, such as a short linker [Asp43–Leu47 (β)] between the “N-stalk” and “β-globule” and the helix-breaking residues [Thr253 (α) or Gly234 (β)} in “antiparallel H5s.” These regions may act as hinges to facilitate domain movement.

### Crystal Structures of the CP/CARMIL Peptide Complexes

**Figure 6:**
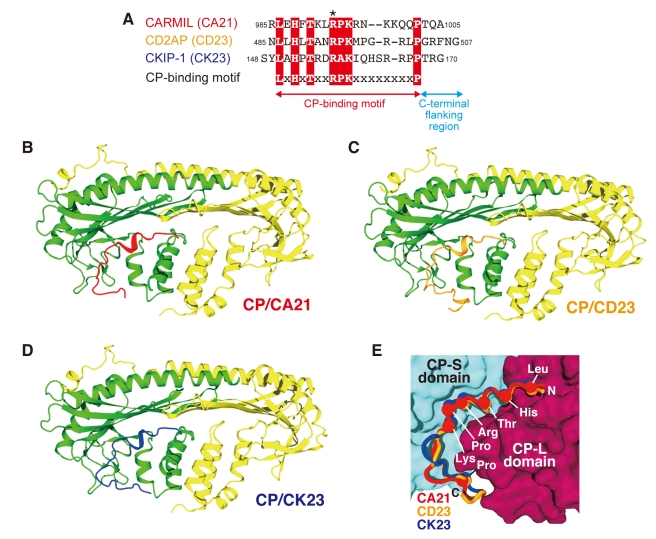
Crystal structures of CP/CARMIL peptide complexes. (A) Sequence alignment of the CARMIL peptides used for crystallization. Conserved residues in the CP-binding motif are highlighted in red. The critical arginine important for the binding of CP is indicated by an asterisk. (B–D) Structures of CP in complex with CA21 (B; red), CD23 (C; orange), and CK23 (D; blue). (E) Superposition of the three peptides. The CP-L (purple) and CP-S (cyan) domains are shown as surface models. The Cα atoms of conserved residues are shown as balls.

To explore the structural basis of CP inhibition by CARMIL proteins, we attempted to determine the structures of CP in complex with CARMIL proteins. Since the CP-binding motif of the CARMIL proteins is sufficient for the interaction with CP [25], peptides derived from this motif were used for the crystallographic studies; mouse CARMIL (residues 985–1005; referred to as CA21), human CD2AP (485–507; CD23), and human CKIP-1 (148–70; CK23) (we collectively refer to these synthetic peptides derived from CARMIL proteins as CARMIL peptides) (Figure 6A). In addition, we chose CP_βΔC_ for crystallization, since the “β-tentacle” does not participate in the CARMIL interaction [23]. All of the crystals were grown under conditions similar to those for the ligand-free CP_βΔC_, and the structures were solved at 1.7–1.9 Å resolutions (*R* = 0.184–0.213, *R_free_* = 0.238–0.263) (Table S1).

The three crystal structures are shown in Figure 6B–D. As expected from the sequence similarity, all three peptides bound to essentially the same surface on CP. A superposition of the three structures further highlights the structural similarity, especially in their N-termini (Figure 6E). In contrast, the C-termini showed some diversity, probably due to the lack of consensus residues and the different peptide lengths. The peptides in our structures are largely unfolded, as previously indicated by a circular dichroism analysis [23]. Each elongated peptide binds along a continuous curved groove on the surface of the CP β-subunit. The peptides are bent by 100° at the conserved proline residue in the middle of the CP-binding motif. The consensus motif interacts with CP across the two domains: the N-terminus with the CP-L domain and the C-terminus with the CP-S domain (Figure 6E). The conformations of CP within the CP/CARMIL peptide complexes are similar to each other (RMSDs; 0.71–0.90 Å) and are slightly different from either CP_full_ or CP_βΔC_ (RMSDs; 0.97–1.26 Å) (Table S3), suggesting that, unlike V-1, the CARMIL peptides do not cause a large conformational change to CP.

**Figure 7:**
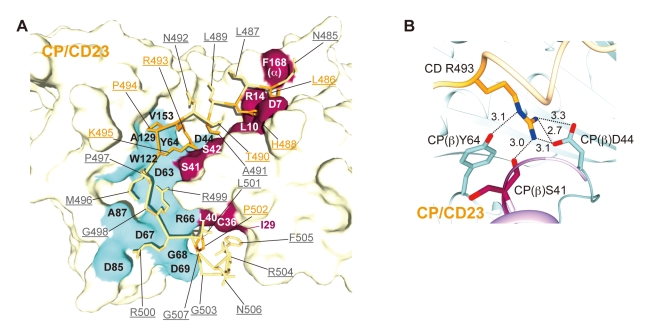
The molecular interface between CP and CARMIL peptides. (A) The intermolecular interactions between residues of CP and CD23. CD23 is shown as a stick model and the labels are underlined. Conserved residues are highlighted in orange. The colored surfaces [CP-L (purple) and CP-S (cyan)} indicate the interface residues. Note that all of the contact residues reside in the CP β-subunit, except for Phe168 (α). (B) Interaction between CD Arg493 and CP β-subunit residues. Salt bridges and hydrogen bonds are indicated by dotted lines with distances in angstroms.

**Figure 8:**
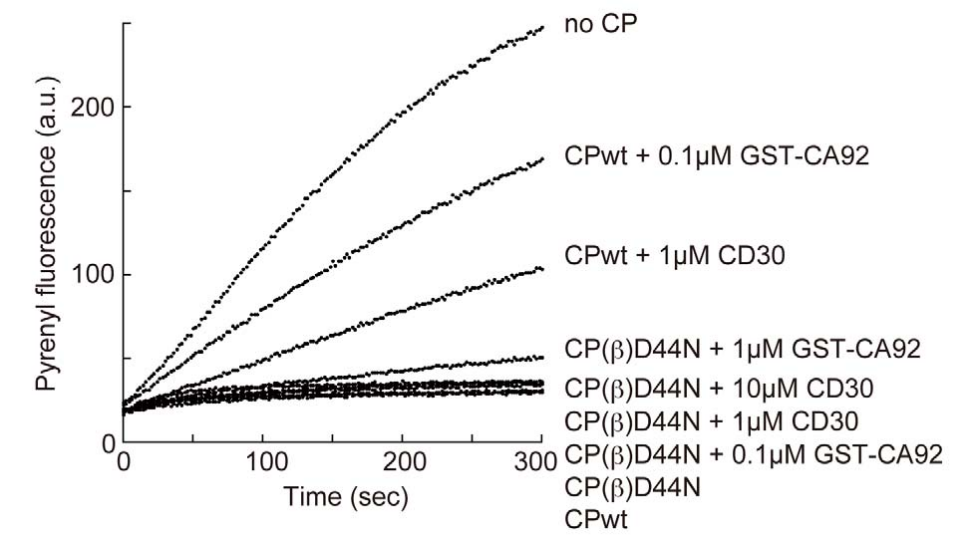
CP (β) D44N is less sensitive to CARMIL proteins. Actin (1.2 µM; 5% pyrene-labeled) was polymerized from spectrin-actin seeds in the presence of 4 nM wild type CP or CP (β) D44N (a mutant CP deficient for CARMIL peptide interaction; see Table 1). CP (β) D44N has identical capping activity to the wild type. As expected, CARMIL constructs cannot effectively prevent the mutant CP from capping actin filaments.

The binding between CP and the CARMIL peptides is primarily mediated by electrostatic interactions, which are supported by hydrophobic interactions (Figures 7A and S5). The mutation of a conserved arginine in the middle of the motif (Arg493 in CD23; indicated by an asterisk in Figure 6A) reportedly abolished CP binding for all of the peptides [23],[25],[31]. This central arginine makes multiple interactions with both the CP-L and CP-S domains, by forming a salt bridge with CP (β) Asp44, and hydrogen bonds with CP (β) Ser41 and Tyr64 (Figure 7B). We confirmed the importance of the intermolecular interface residues of CP by biochemical assays using mutant CP proteins (Figure 8 and Table 1). Among the mutant CP proteins, CP (β) D44N exhibited the lowest affinity for the CARMIL peptides.

**Table 1:** Binding affinities between mutant CPs and CARMIL proteins fragments.

Immobilized/Analyte	K_on_ (M^−1^s^−1^)	K_off_ (s^−1^)	K_D_ (nM)
GST-CD56/CP (β) D44N^b^	—	>0.1	16,000
GST-CD56/CP (β) D63N^b^	—	>0.1	1,200
GST-CD56/CP (β) Y64F^a^	9.9×10^5^	5.2×10^−3^	5.2
GST-CD56/CP (β) D67N^b^	—	>0.1	8,500
GST-CA92/CP (β) D44N^b^	—	>0.1	7,400
GST-CA92/CP (β) D63N^b^	—	>0.1	1,400
GST-CA92/CP (β) Y64F^a^	4.6×10^5^	4.6×10^−3^	10
GST-CA92/CP (β) D67N^b^	—	>0.1	4,900

a K_D_ values were calculated from the kinetic rate constants (K_D_ = k_off_/k_on_).

b K_D_ values were obtained from Michaelis-Menten plots under saturated binding conditions.

### The C-Terminal Flanking Region of the CP-Binding Motif Is Required for High Affinity CP Binding

**Figure 9:**
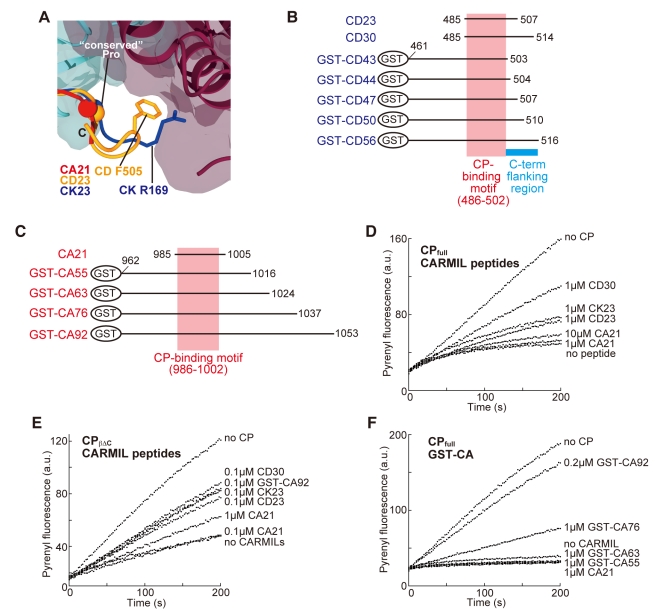
The importance of the C-terminal flanking region of the CP-binding motif. (A) Superposition of the C-termini of the peptides. CD Phe505 and CK Arg169 are shown as stick models. The conserved proline at the C-terminus of the motifs is shown as balls. (B) CD2AP constructs used for biochemical assays. (C) CARMIL constructs used for biochemical assays. (D–F) The effect of CARMIL fragments on the barbed end capping activity of CP was measured for the ability to increase the actin elongation rate in the presence of CP. Actin (1.2 µM, 5% pyrene-labeled) was polymerized from spectrin-actin seeds in the presence of 3.6 nM CP_full_ (D), 10 nM CP_βΔC_ (E), or 7.2 nM CP_full_ (F) with various concentrations of CARMIL peptides or GST-CA constructs.

In addition to their extensive interactions through the CP-binding motif, CD23 and CK23 further associate with the CP “N-stalk” via the C-terminal flanking residues of the motif. In the CP/CD23 complex, CD Phe505 contacts the hydrophobic pocket formed by the CP “N-stalk” residues [CP (β) Ile29, Cys36, and Leu40] and the peptide residues (CD Leu501 and Pro502) (Figure S6A). In the CP/CK23 complex, the C-terminal residue of the peptide, CK Arg169, forms an electrostatic interaction with CP (β) Asp30 (Figure S5B). In contrast to these two peptides, CA21 does not contact CP via the C-terminal flanking region (Figures 9A and S5A).

We tested the importance of the C-terminal flanking regions of the CP-binding motif using a binding assay (Table 2; the constructs used for the measurement are shown in Figure 9B). Surprisingly, GST-CD43, lacking CD Phe505 but containing the entire consensus motif, bound to CP only weakly with a K_D_ of 260 nM, suggesting that the CP-binding motif of CD2AP alone is not sufficient for stable interaction with CP. In contrast, longer constructs with extended C-terminal residues showed higher CP binding affinities than the shorter fragments. GST-CD47, containing CD Phe505, bound to CP with a K_D_ of 18 nM and GST-CD56 bound tightly to CP (K_D_ = 4.7 nM), in good agreement with the previously reported value (K_D_ = 5.6 nM for GST-CD2AP fragment containing residues 474–513 [25]). The C-terminus of CD23 extends into the region between the CP-L and CP-S domains (Figure S6B). Thus, the residues immediately C-terminal to CD23 (i.e., CD Gly508∼) are expected to form additional contacts with the domain boundary residues to stabilize the CP/CD2AP complex. Collectively, the C-terminal flanking region of the consensus motif is required for the stable interaction between CP and CD2AP.

**Table 2:** Binding affinities between CP and CARMIL peptides.

Immobilized	Residues	k_on_ (M^−1^s^−1^)	k_off_ (s^−1^)	K_D_ (nM)
GST-CD43	461–503	—	>0.1	260^a^
GST-CD44	461–504	5.4×10^5^	0.033	62^b^
GST-CD47	461–507	1.6×10^6^	0.029	18^b^
GST-CD50	461–510	1.2×10^6^	0.018	15^b^
GST-CD56	461–516	1.3×10^6^	5.9×10^−3^	4.7^b^
GST-CA55	962–1016	—	>0.1	4,000^a^
GST-CA63	962–1024	—	>0.1	3,300^a^
GST-CA76	962–1037	2.3×10^5^	0.019	80^b^
GST-CA92	962–1053	1.6×10^6^	5.2×10^−3^	3.3^b^

a K_D_ values were calculated from the kinetic rate constants.

b K_D_ values were obtained from Michaelis-Menten plots under saturated binding conditions.

We also examined GST-CARMIL fragments (Table 2 and Figure 9C). Both GST-CA55 and GST-CA63, containing the entire CP-binding motif and 10 or more extra residues at either end, bind only to CP with K_D_s in the micromolar range. This confirms that the consensus motif alone cannot tightly bind to CP. Moreover, unlike CD2AP, the CARMIL residues immediately C-terminal to the motif do not contribute to the stable CP interaction, consistent with our structure in which CA21 does not contact CP in this region. The stable CP interaction was observed in longer CARMIL fragments. GST-CA76 was found to have modest binding affinity to CP (K_D_ = 80 nM) and GST-CA92 bound strongly to CP (K_D_ = 3.3 nM) and with a comparable K_D_ to GST-CD56.

We next evaluated the CP-binding affinity of CK23 by a competition assay and found that both CD23 and CK23 effectively compete with immobilized GST-CA92 for CP binding, whereas CA21 was a less efficient competitor (Figure S7). Thus, CK23 appears to have CP binding affinity comparable to CD23.

The CP binding affinity of the CARMIL peptides directly correlated with their ability to inhibit the barbed end capping. CD23 and CK23 moderately inhibited barbed end capping by CP, while CA21 was a poor inhibitor (Figure 9D). Furthermore, CD30, a peptide with 7 extra residues at the C-terminus of CD23, showed higher CP inhibition activity than CD23 (Figure 9D). Although weaker than CD23 or CK23, CA21 retained the ability to inhibit CP, since CA21 attenuated the barbed end capping by CP_βΔC_ (Figure 9E), which is a less potent capper compared to CP_full_ [4]. Intriguingly, all peptides tested effectively inhibited CP_βΔC_, suggesting that CARMIL peptides do not inhibit CP simply by preventing the “β-tentacle” from filament binding. We next tested the CP inhibitory activity of GST-CARMIL constructs. As expected from their CP binding affinities, GST-CA92 showed the strongest CP inhibitory effect (Figure 9F). GST-CA92 appears to have full CP inhibition activity, because it showed a similar level of inhibition as GST-C-1 (residues 962–1084), which has the same activity as the full length CARMIL (unpublished data [23]).

### CARMIL Peptides Do Not Sterically Inhibit CP

**Figure 10:**
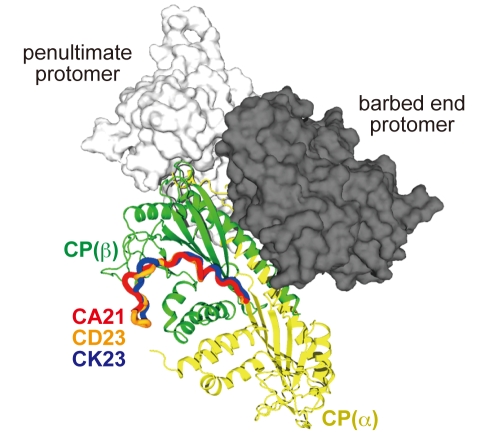
CARMIL peptides do not directly compete with the barbed end for CP binding. Superposition of the CP/CARMIL peptide complex structure on the EM model of the CP/barbed end structure using the Cα positions of CP (β) 3–244. The barbed end (gray) and the penultimate (white) protomers are shown as surface models.

A superposition of the crystal structures of the CP/CARMIL peptide complexes onto the EM model of the CP/actin filament structure clearly revealed that none of the peptides on CP overlap with the barbed end actin protomers (Figure 10). As described above, all of the peptides used for the crystallization have varying degrees of CP inhibition activity (Figure 9D–F). Furthermore, the C-terminal flanking residues of CD23, which greatly contribute to the CP inhibition, cannot reach the nearest surface of the actin filament. Therefore, unlike V-1, the CARMIL peptides do not inhibit the barbed end capping activity of CP by steric hindrance.

This non-overlapping CP interaction, permitting the CARMIL peptides to interact with the filament-bound CP, is a prerequisite for the uncapping activity. Furthermore, the “α-tentacle” including the “basic triad” on the top surface of CP, the primary actin binding site, is still exposed even when CP is bound with CARMIL proteins. This allows the CP/CARMIL protein complex to make an initial contact with the barbed end, and thus CARMIL proteins cannot sequester CP completely from the barbed end.

### CARMIL Peptides Allosterically Inhibit CP/V-1 Binding

**Figure 11:**
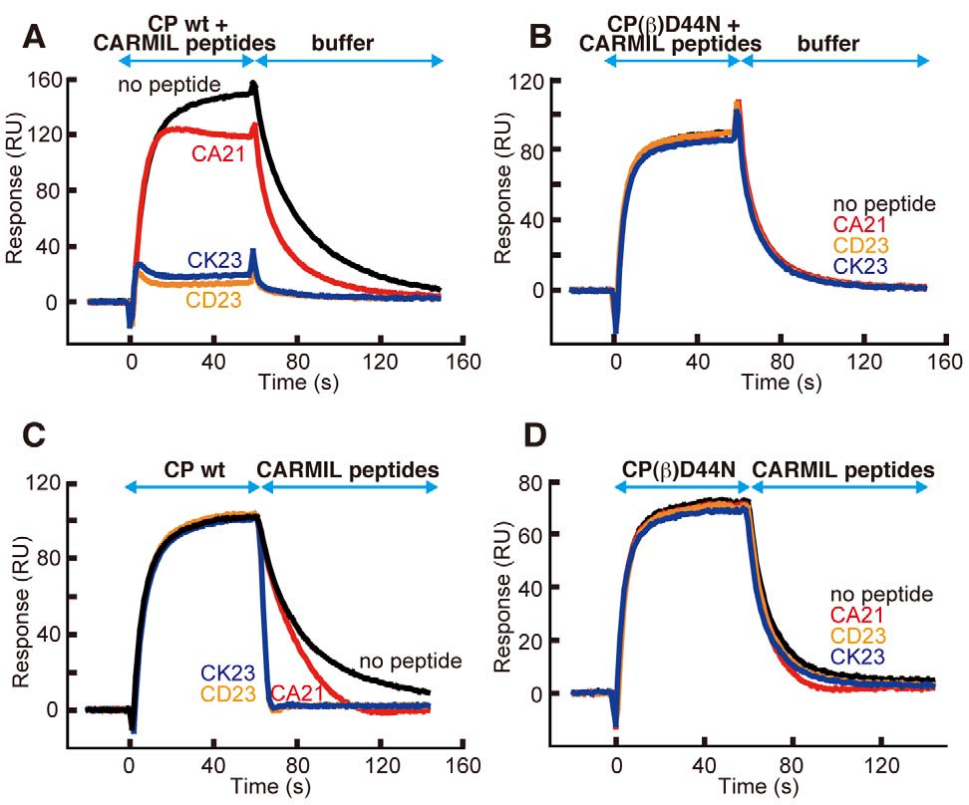
CARMIL peptides allosterically inhibit CP/V-1 binding. The ability of CARMIL peptides to inhibit CP binding to V-1 was tested by a surface plasmon resonance assay. GST-V-1 was immobilized on a sensor chip. (A and B) Mixtures of 100 nM CARMIL peptides and 45 nM CP wt (A) or CP (β) D44N (B) were perfused to assess the effect of the peptides on the formation of the CP/V-1 complex. (C and D) Initially, 45 nM CP wt (C) or CP (β) D44N (D) was perfused to form the GST-V-1/CP complex, and subsequently 100 nM CARMIL peptides were perfused to observe the effect of the peptides on the dissociation of CP from the immobilized GST-V-1.

The CP binding site of V-1 is located on an opposite face from the CARMIL peptide binding site, implying that CP can simultaneously bind both inhibitors. Conversely, we found that the conformation of CP_V-1_ is significantly different from that of the CARMIL peptide-bound CP (CP_CARMILs_) (Table S3), because the binding of V-1 induces a twisting movement of the CP-L and CP-S domains. This raises the possibility that the CARMIL peptides allosterically inhibit CP from binding V-1 by restricting the domain twisting, since the peptides bind to CP across the two domains. We tested this prediction using a surface plasmon resonance assay. We immobilized GST-V-1 on a sensor chip, and then perfused with CP premixed with CARMIL peptides. Surprisingly, CD23 and CK23, which possess substantial affinity for CP, strongly inhibited the CP/V-1 interaction, indicating that the peptides restrict the conformation of CP to the “low affinity to V-1” form (Figure 11A). This inhibition depends on the CP/CARMIL peptide interaction because CA21, which has a lower CP binding affinity than the other peptides, exhibited minimal inhibition (Figure 11A). Furthermore, none of the peptides tested could prevent CP (β) D44N, a mutant CP deficient in CARMIL protein interaction (Table 1 and Figure 8), from the V-1 interaction (Figure 11B). Most notably, in addition to its effect on free CP, the CARMIL peptides can act on CP pre-bound to V-1 and facilitate the dissociation of the complex. When the preformed CP/V-1 complex bound on the sensor chip was perfused with CD23 or CK23, CP dissociated from V-1 quite rapidly, as compared with the buffer control (Figure 11C). Again, we found that CA21 was less effective in facilitating the dissociation (Figure 11C), and that the interaction between CP (β) D44N and V-1 was not affected by CARMIL peptides (Figure 11D). This result suggests that the CARMIL peptides possess the ability to interact with CP in a conformation different from CP_CARMILs_ and to shift the CP conformation toward the CP_CARMILs_ form.

We further confirmed the effect of the CARMIL peptides on CP/V-1 interaction by a pull-down assay. Under equilibrium conditions, the binding of CP to GST-V-1 was inhibited by the addition of the peptides in a concentration-dependent manner (Figure S8). Collectively, we concluded that the CARMIL peptides allosterically inhibit CP binding to V-1.

### CARMIL Peptides Change the Intrinsic CP Fluctuation

**Figure 12:**
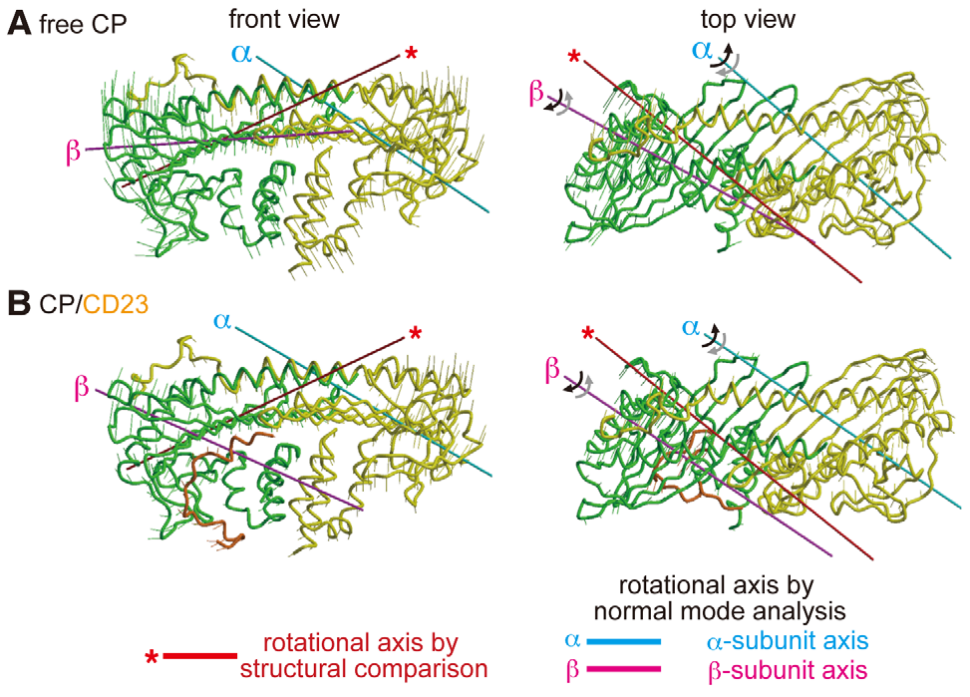
Normal mode analysis supports the intrinsic flexibility of the CP molecule. Free CP (A) and the CP/CD23 complex (B) are shown. Two rotational axes of the first mode, which run through the α- (cyan) and β-subunits (magenta), respectively, are presented. The directions of the motion are indicated by black and gray sets of arrows. Whiskers indicate the relative amplitudes of positional fluctuations of Cα associated with the first slowest mode. Red rods with an asterisk indicate the rotational axis of the twist movement between the CP-L and CP-S domains, determined from a comparison of the crystal structures.

To further explore the intrinsic flexibility of the CP molecule, we performed a normal mode analysis with an elastic network model (ENM). In this model, a protein is considered as a simple elastic object, and the spatially neighboring residues in the native structure are connected by Hookian springs. Based on this approximation, the intrinsic fluctuations originating from the protein shape are revealed. The normal mode analysis on the ENM has been applied to various sizes of proteins, e.g., lysozyme [32], F1-ATPase [33], and chaperonin GroEL [34]. Referring to the lower frequency modes, the analysis succeeded in reproducing large conformational motions that had been experimentally revealed [35]. We applied this method to the CP/CD23 complex (Figure 12A) and the CP structure extracted from the complex (Figure 12B), and focused on the first lowest modes. The first lowest mode of CP can be described as twisting motions relative to two axes, which run through the α- and β-subunits, respectively (Figure 12A and Table S4; see Materials and Methods for more details). In this mode, the directions of the twisting movements about the two axes are opposite from each other (indicated by black and gray sets of arrows in Figure 12). Among these two axes, the β-subunit axis almost coincides with the axis of the twist movement between the CP-L and CP-S domains that was revealed by the structural comparison (red rods with asterisk in Figure 12). This finding strengthens the notion that CP continually undergoes substantial twisting movements about this axis. Furthermore, we found that the CARMIL peptides alter this intrinsic mode, both in the direction of the rotational axis and the amplitude of the motion (Figure 12B). These effects are observed almost exclusively in the twisting motion about the β-subunit axis, yet not about the α-subunit axis, suggesting that the CARMIL peptide suppresses the twisting movement between the CP-L and CP-S domains.

## Discussion

### Mechanism of CP Regulation by V-1

**Figure 13:**
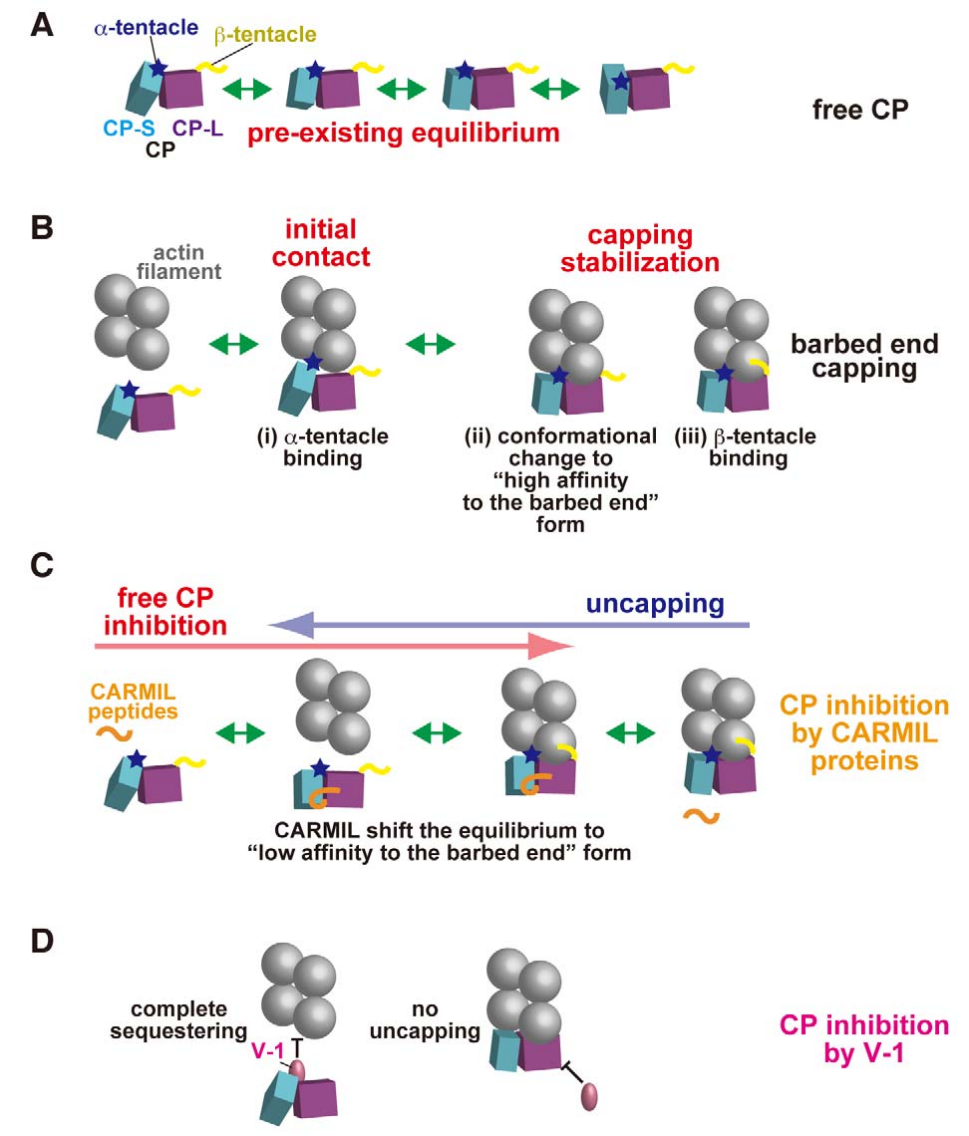
Model for the filament capping by CP and its inhibition by V-1 and CARMIL proteins. (A) Free CP is in equilibrium between pre-existing multiple conformations, which can be attributed to the twisting movement between the CP-L (purple) and CP-S (cyan) domains. The affinity of CP for the barbed end is dependent on its conformation. (B) The barbed end capping by CP. (i) “Basic triad” residues on the CP “α-tentacle” region (blue star) interact electrostatically with the barbed end. This initial contact is further stabilized by (ii) an adaptive conformational change to a “high affinity to the barbed end” form and (iii) a supportive binding of the “β-tentacle” (yellow) to the filament. (C) CARMIL proteins allosterically inhibit CP by disturbing its conformational flexibility [i.e., preventing step (ii) in (B)}. In free CP inhibition (red arrow) CARMIL proteins bind to CP across the two domains, thus restraining the twisting motion. In uncapping (blue arrow), CARMIL proteins interact with the barbed end-bound CP. This is possible because the binding site is not hindered by the actin protomers. In either process, CARMIL proteins shift the conformational equilibrium of CP toward the “low affinity to the barbed end” form, thereby attenuating the capping activity. Note that the CP bound CARMIL proteins do not directly affect the α- or β-tentacle interactions to the filament. (D) In contrast to CARMIL proteins, V-1 sterically hinders CP from the barbed end by interacting with its primary actin binding site, thereby abolishing the filament capping activity [i.e., step (i) in (B) is inhibited}. Simultaneously, V-1 lacks uncapping activity, because the V-1 binding site on CP is buried deeply between the two end protomers when CP caps the filaments. Note that V-1 binding must affect the conformational flexibility of CP, since it holds CP in the CP_V-1_ conformation. However, this effect appears not to be the main cause of CP inhibition by V-1.

The crystal structure of the CP/V-1 complex revealed that V-1 mainly interacts with the “α-tentacle,” the primary actin binding surface of CP, thereby sterically hindering CP from barbed end capping (Figures 1–3). The structure supports biochemical data that V-1 has no uncapping activity (Figure 13D). A sequence alignment of V-1 indicates that the residues involved in the V-1 interaction are highly conserved through evolution, despite their relatively minor contributions to the protein fold (Figure S9). Furthermore, the “basic triad” in the CP α-subunit, containing the highly conserved residues critical for actin binding is also recognized by V-1. This suggests that the architecture of the V-1 molecule is well suited for the interaction with CP, i.e., CP inhibition is the key role for V-1 in various cellular processes. This notion is further supported by the finding that, in cultured cells, V-1 is involved in the regulation of actin assembly and cell morphology (Figure 4). We note that CARMIL peptides inhibit CP from binding V-1 (Figures 11 and S8), indicating that the effect of V-1 on CP may be under the control of other proteins which interact with CP or V-1. Future studies will verify the role of V-1 in actin-driven cell motility.

### Conformational Flexibility of CP and the Barbed End Capping

An unexpected finding in this study was the conformational flexibility of the CP molecule. A structural comparison analysis revealed that CP consists of two rigid domains, CP-L and CP-S, and undergoes conformational changes even in the absence of a ligand (Figure 5). This intrinsic twisting motion between the two CP domains was further supported by a normal mode analysis of free CP (Figure 12A). Intriguingly, our analysis also predicts that, in addition to the domain twist related to the rotational axis passing through the β-subunit, there might be an analogous twisting movement about the α-subunit axis. This is plausible because CP has pseudo 2-fold rotational symmetry [3]. Thus, the CP-L domain might be further divided into two rigid subdomains, which also undergo a twisting movement relative to each other.

Our data showed that the CP-binding motif of CARMIL proteins cannot bind tightly to CP, despite the multitude of intermolecular interactions present in the structures (Figures 7, 9, S5, and Table 2). This is attributable to the conformational fluctuation of CP, as the consensus motif interacts with residues at the domain boundary that may act as a hinge in the twisting movement. We demonstrated that the regions C-terminal to the CP-binding motif are responsible for the strong interactions between CP and CARMIL proteins (Table 2). Thus, the consensus motif and the flanking region may reciprocally increase their affinity for CP, which in turn would inhibit CP effectively.

The tight interaction between CP and the barbed end is contributed by the extensive inter-molecular surface residues [5]. Consequently, the intrinsic twisting motion between the two CP domains that can cause changes in the overall structure must affect the capping activity of CP. Therefore, for a stable filament capping, CP accommodates its shape to a favorable conformation for the barbed end interaction. Consequently, we have revised the previous two-step capping model [5] as follows: (i) “Basic triad” residues on the CP “α-tentacle” region interact electrostatically with the barbed end. This initial contact is followed by two independent stabilization steps: (ii) an adaptive conformational change to a “high affinity to the barbed end” form that is a twisting movement between the CP-L and CP-S domains and (iii) the supportive binding of the “β-tentacle” to the filament (Figure 13B). Hence, a factor which disturbs either of the capping steps has an inhibitory effect on the filament capping activity of CP. For example, V-1 sterically hinders CP from the barbed end by blocking step (i).

### CARMIL Proteins Allosterically Inhibit CP by Suppressing Its Conformational Fluctuations

How do CARMIL proteins inhibit the capping activity of CP in an allosteric manner? We showed that CARMIL peptides allosterically inhibit the interaction of CP with V-1 (Figures 11 and S8). This finding indicates that, regardless of the initial CP state (i.e., free or V-1-bound), the peptides binding across the two CP domains shift the conformational distribution to within a narrow range around CP_CARMILs_, conformations that are unfavorable for V-1 binding. We propose that CARMIL proteins inhibit CP in a similar manner (Figure 13C); CARMIL proteins limit the conformational distribution of CP to mostly the “low affinity to the barbed end” form, leading to attenuation of the barbed end capping activity [i.e., step (ii) in Figure 13B is inhibited]. Fujiwara et al. indicated that CARMIL does not affect the association of CP to the barbed end but accelerates its dissociation from the filament since the on rate of the CP/CARMIL complex to the barbed end is virtually the same as that of free CP (3.7 µM^−1^s^−1^ versus 2.6 µM^−1^s^−1^), while the affinity of the complex to the filament is significantly lower than that of free CP (K_D_ = 38 nM versus 0.18 nM)[29]. This is consistent with our hypothesis that the CARMIL proteins inhibit CP only by affecting the twisting motion which provides the capping stability, since our data showed that neither the “α-tentacle” (the capping on rate determinant) nor the “β-tentacle” (the other capping stabilizer) is disturbed by the CARMIL protein. Furthermore, our prediction that the conformation CP_CARMILs_ is substantially different from the “high affinity to the barbed end” form is consistent with the concept that CARMIL binding to free CP must involve some surface or conformation that is not available when CP is bound to a barbed end [23]. This is because the affinity of CARMIL for the barbed end-bound CP has been estimated to be 10- to 100-fold [23] or 200-fold [29] lower than that for free CP.

To better understand the mechanism of CP inhibition by the CARMIL proteins, it would be helpful to know the conformation of CP on the barbed end. As such, we fitted all known crystal structures of CP to the 3D electron density map of the CP/actin filament [5] and found that all of the structures tested fit similarly to the model except for CP_V-1_, which did not fit as well (Figure S10). The mismatch between the EM envelope and CP_V-1_ is largely due to the shift of the CP-S domain relative to the CP-L domain, suggesting that the CP in the “high affinity to the barbed end” form may not adopt such an “open” conformation as in CP_V-1_.

In this study, we cannot provide structural information about CP bound to the full activity CARMIL fragments. During the submission of this manuscript, Robinson and colleagues reported a crystal structure of CP in complex with a CARMIL fragment with an extended C-terminal portion (CBR115; human CARMIL residues 964–1078) [36]. This structure revealed that, in addition to the CP-binding motif, a 15 residue motif serves as a second CP binding site (CARMIL-specific interaction motif, residues 1021–1035; highlighted by orange in Figure S11). The motif binds to the CP “N-stalk” in the CP-L domain, on the side opposite to where the CP-binding motif binds. This result also supports the concept that CARMIL proteins inhibit CP in an allosteric manner (see Text S1 for a detailed discussion about the role of the C-terminal flanking region of the CP-binding motif of the CARMIL proteins for CP inhibition).

Recently, intrinsically unstructured proteins or segments of proteins have been recognized to play critical roles in many cellular processes such as transcriptional regulation and signal transduction [37]. These disordered regions usually fold into ordered secondary or ternary structures upon binding to their targets (termed coupled folding and binding processes). We revealed, however, that the CARMIL peptides are functional in suppressing the conformational flexibility of CP, although they have an extended backbone conformation. Consequently, our results provide new insights into the functional expression of intrinsically unstructured proteins.

### Implications for Dynamic CP Behavior in Cells

An important implication of this study is that conformational restraints placed on CP lead to an attenuated affinity of the protein for the barbed end. This raises the possibility that other CP regulators, such as PIP_2_, also modulate the capping activity. Moreover, the state of the actin filament would also affect the affinity of CP towards the filament; i.e., a certain actin binding protein that changes and/or restricts the structure of the barbed end to an unfavorable form for CP binding can antagonize the filament capping. We assume that such a mechanism may account for the rapid turnover rate of CP in lamellipodia [9],[10].

In this study, we have described the structural basis for CP inhibition by two regulators, V-1 and CARMIL proteins. Our findings suggest that CP is not a constitutively active inhibitor of barbed end elongation; rather, the capping activity of CP is fine-tuned for the highly orchestrated assembly of the cellular actin machinery, and the conformational flexibility of CP provides the structural basis for the regulation.

## Materials and Methods

### Proteins

Expression vectors for chicken CP_full_ and CP_ΔβC_ were constructed in pETDuet-1 by PCR, using pET-3d/CP [38] as the template. CP was expressed in *E. coli* Rosetta2 (DE3) and was purified as described [3]. V-1 (human), expressed in *E. coli* Rosetta2 (DE3) as a GST-fusion protein, was affinity-purified and the tag was removed. Synthetic peptides derived from CARMIL proteins were obtained from Invitrogen. For crystallization, CP was incubated with a 1.2–2.0-fold molar excess of V-1 or CARMIL peptides at 4°C for 2 h, followed by gel filtration to purify the complexes. Expression vectors for the GST-CA constructs were prepared from the mouse cDNA clone as previously described [23]. Vectors for GST-CD fragments were constructed by PCR cloning using a human whole brain cDNA library (Clontech) as the template. Amplified DNA fragments were cloned into pGEX-6P-1. GST-fusion proteins were expressed in *E. coli* Rosetta2 (DE3) cells and affinity-purified using glutathione sepharose resin. Mutations were introduced using a Quikchange mutagenesis kit (Stratagene). Actin was prepared from rabbit skeletal muscle, as previously described [39], and was further purified by gel filtration chromatography. Pyrene labeled-actin was prepared as described [40]. Spectrin-actin seeds were prepared from rabbit red blood cells, as previously described [41].

### Crystallization, Data Collection, and Structure Determination

Each protein complex, at 8–10 mg/ml in 1 mM DTT and 5 mM Tris-HCl (pH 8.0), was mixed with an equal volume of reservoir solution as follows: 10% PEG4000, 20% isopropanol, 20 mM EDTA, 0.1 M Tris-HCl (pH 8.4) for CP/V-1; 12.5% PEG400, 20 mM BaCl_2_, 0.1 M MES-NaOH (pH 6.0) for CP_βΔC_; 18% PEG400, 40 mM BaCl_2_, 0.1 M MES-NaOH (pH 6.0) for CP/CA21; 10% PEG400, 20 mM BaCl_2_, 0.1 M MES-NaOH (pH 6.5) for CP/CD23; and 17.5% PEG400, 30 mM BaCl_2_, 0.1 M MES-NaOH (pH 6.0) for CP/CK23. The crystals were grown at 20°C by the hanging-drop vapor diffusion method and were cryoprotected with their reservoir solutions supplemented with 20% glycerol (for CP/V-1) or with 35% PEG400 (for other crystals) prior to flash-cooling in a cold nitrogen stream. Diffraction data were collected in the BL26B1 beamline at SPring-8 [42] and were processed with HKL2000 [43]. Space groups and cell parameters are listed in Table S1. Initial phases were determined by molecular replacement with Molrep [44], using the CP structure as a search model. Model building and refinement were performed with CNS [45], Refmac [46], and Coot [47]. Each crystal contains one CP or CP/inhibitor complex in the asymmetric unit. Data collection and refinement statistics are summarized in Table S1.

### Actin Polymerization Assay

The barbed end elongation assay from spectrin-actin seeds was performed essentially as previously described [4]. Briefly, G-actin was stored in G-buffer (0.2 mM CaCl_2_, 0.2 mM ATP, 0.5 mM DTT and 10 mM imidazole, pH 7.0). At 90 s prior to polymerization, the Ca^2+^ was replaced with Mg^2+^, by the addition of 1/10 volume of 10 mM EGTA and 1 mM MgCl_2_ to G-actin. Barbed end elongation was initiated by mixing the solutions in the following order: Mg^2+^ actin (5% pyrene-labeled), CP, V-1 or CARMIL protein, a 1/20 volume of 20× polymerization buffer (1 M KCl, 20 mM MgCl_2_, 20 mM EGTA, 0.2 M imidazole, pH 7.0) and spectrin-actin seeds. Actin polymerization was measured by monitoring the pyrene-actin fluorescence (excitation 370 nm; emission 410 nm) at 25°C.

### Surface Plasmon Resonance Measurements

The binding affinities of CP for V-1 or CARMIL proteins were evaluated by surface plasmon resonance measurements with Biacore 3000 or Biacore 2000 instruments (GE Healthcare). GST-fusion proteins (GST-V-1, GST-CA, or GST-CD) were immobilized onto a CM5 sensor chip up to 200 RU (response units; 200 pg/mm^2^) via anti-GST antibodies. CP at various concentrations in running buffer (50 mM KCl, 1 mM MgCl_2_, 0.005% Tween-20, 10 mM imidazole, pH 7.0) was perfused over the chip at 20°C, at a flow rate of 20 µl/min. Response curves were obtained by subtracting the background signal generated simultaneously on a control flow cell with immobilized GST. To measure the effect of the CARMIL peptides on the facilitation of CP/V-1 dissociation (in Figure 11C and 11D), we used the “co-inject” mode for successive injections of the peptides followed by CP. Kinetic parameters were determined by fitting the sensorgrams to a simple 1∶1 binding model, using the Bia-evaluation software (GE Healthcare). K_D_ values were obtained from the kinetic rate constants. For several mutant proteins possessing fast dissociation rates for the ligand (k_off_ >0.1 s^−1^), we measured the amount of bound-CP at the steady state over a wide concentration range. K_D_ values were evaluated by plotting these values against the concentrations of CP.

### Cultured Cell Analysis

The stable V-1 overexpression transfectant (V1-69) and its mock transfectant (C-9), established in the PC12D subclone of rat pheochromocytoma cells, were cultured as reported previously [16]. The concentrations of F- and G-actin were measured using an assay kit (Cytoskeleton), as described previously [48]. For subcellular fractionation, the cells were homogenized by sonication in homogenization buffer (150 mM NaCl, 2 mM EGTA, 10 mM Tris-HCl, pH 7.4, with protease inhibitors). The extracts were centrifuged at 100,000 *g* for 60 min, and the supernatant was designated as the “high speed supernatant” fraction. The pellet was incubated for 30 min in the homogenization buffer supplemented with 0.5% Triton X-100 and ultracentrifuged. This supernatant was designated as the “high speed pellet soluble in detergent” fraction, and the “high speed pellet insoluble in detergent” fraction was obtained by further extraction of the pellet in 8.3 M urea. The amount of CP in the fractions was determined by Western blotting with an anti-CP β-subunit antibody [21]. For morphological analysis, cells cultured at a density of 5×10^4^ cells per well on the poly-d-lysine-coated culture slides (BD Biosciences) for 24 h were fixed by 3.7% formaldehyde in PBS and permeabilized with 0.1% Triton X-100 in PBS. Fixed cells were pre-incubated with the Image-iT FX signal enhancer (Invitrogen) and counter-stained with Alexa Fluor 546-conjugated phalloidin (Invitrogen) and Hoechst 33258 (Dojin). The fluorescence images were obtained using Leica microfluorescent system (AF6500; Leica Microsystems).

### Normal Mode Analysis by the ENM

The intrinsic flexibility of CP was examined by the normal mode analysis with the ENM [49],[50],[51]. In this model, only the Cα atoms are considered, and a harmonic potential with a single parameter, *C*, is introduced between all Cα atoms within a cut-off distance, 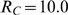 Å. The potential energy of a protein is given as
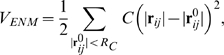
where 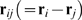 is the vector connecting the *i*-th and *j*-th Cα atoms and 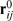 is that in the crystal structure. The Hessian matrix, whose elements are the second derivatives of the potential energy, was derived and diagonalized, and we obtained the eigenvectors and eigenvalues, representing the normal modes.

Since the twisting movements were revealed by comparisons of the crystal structures, we estimated the intrinsic rotations from the lowest frequency mode that corresponds to the largest vibration. As the CP free model structure, we employed the CP structure of the CP/CD23 complex (i.e., the CD2 peptide was removed). The displacements of each Cα atom were derived from the displacement vector, the eigenvector of the lowest frequency mode scaled by the reciprocal of the eigenvalue. We consider that the set of Cα atoms with small displacements represents the rotation axis. The Cα atoms, whose squares of the displacements were smaller than 2 Å^2^, were collected.

We found that these Cα atoms could be clearly divided into two groups, and each of them was separately distributed in the α-subunit or the β-subunit (Table S4). The coordinates of these Cα atoms in each group were evaluated by the principal component analysis, and the first components defined the rotation axes on the α- and β-subunits. In Figure 12, the axes run on the center of Cα atoms with small displacements. The same analysis was applied to the CP/CD23 complex, with a cut-off displacement of 1 Å^2^.

### Accession Numbers

The Protein Data Bank accession codes for the crystal structures determined in this study are as follows: CP/V-1 (3AAA), CP_βΔC_ (3AA7), CP/CA21 (3AA0), CP/CD23 (3AA6), and CP/CK23 (3AA1).

## Supporting Information

Figure S1 **Primary sequence of CP.** The amino acid sequences of the chicken CP α1-subunit (A) and β1-subunit (B) are shown. Bars and arrows above the sequences represent α-helices and β-strands, respectively; asterisks and exclamation marks indicate the V-1 and CD23 interacting residues, respectively. The loop S5–S6 (β), which is important for V-1 binding, is indicated as a green wavy line. Residues in the CP-S domain are highlighted in cyan. Each structural motif is indicated with a bar underneath, in the colors corresponding to those in Figure 1A. At the N-terminus, each CP subunit has three α-helices (we call this region the “N-stalk”). On each side of the “N-stalk,” short β-strands are packed in a unique manner to form globular structures that are flanked by helix 4 of either subunit (“α-globule” and “β-globule”). A large anti-parallel β-sheet consisting of 10 β-strands forms the central layer (“central β-sheet”). Helix 5 of both subunits, containing a helix-breaking residue [Thr253 (α) and Gly234 (β)], lies above the “central β-sheet” in an anti-parallel fashion (“antiparallel H5s”), and each is flanked by C-terminal extensions that possess barbed end capping activity (“α-tentacle” and “β-tentacle”).
(0.39 MB TIF)

Figure S2 **V-1 mutants deficient in CP-binding fail to inhibit CP's capping activity.** Actin (1.2 µM; 5% pyrene-labeled) was polymerized from spectrin-actin seeds in the presence of 3 nM wild type CP with various concentrations of wild type V-1 or mutant V-1 proteins. The addition of wild-type V-1 to the system at a concentration well above the K_D_ inhibited CP from barbed end capping. In contrast, the CP-binding deficient V-1 mutants (V-1 W8A, D44R, or E78R; see Table S2) showed minimal effects on CP capping.
(0.16 MB TIF)

Figure S3 **Crystal structure of CP_βΔC_.** Crystal structure of CP_βΔC_ (chicken α1 full/β1 1–244; a deletion mutant CP lacking the “β-tentacle”) at 1.9 Å resolution. Two orthogonal views of the structure are shown in ribbon models with the α-subunit (yellow) and β-subunit (green). The N- and C-termini are indicated. The secondary structures of CP_βΔC_ are nearly identical to those of CP_full_, although the overall conformations are significantly different between the two structures (see Table S3).
(1.04 MB TIF)

Figure S4 **Structural change to CP induced by V-1 binding.** The C-terminal region of the CP α-subunit (residues 243–275) and the loop (β) S5–S6 are shown in ribbon models. CP_full_: CP-L (white) and CP-S (orange): CP_V-1_: CP-L (gray) and CP-S (purple); V-1: magenta. Molecular interface residues [CP (α) Lys256, Arg260 and Arg266, V-1 Trp8, Asp44 and Glu78] are shown as stick models and hydrogen bonds are indicated by green lines. Shifts of CP_V-1_ induced by the V-1 interaction are represented by cyan arrows. Upon V-1 binding, the CP-S domain rotates by approximately 10° relative to the CP-L domain about a rotation axis that is nearly identical to the long axis of the molecule (see Figure 5F and 5G). Since V-1 Glu78 interacts simultaneously with CP (α) Lys256 near the domain boundary and with Arg266 in the “α-tentacle,” it pulls Arg266 towards Lys256 by ∼2.7 Å (Cα positions). This shift straightens the “antiparallel H5s” and further moves the rest of CP-S bound tightly with the “α-tentacle.” As a result, the distance between the “N-stalk” and “β-globule” becomes wider (see Figure 5A and 5B). Simultaneously, V-1 ANK 1 pushes down the CP (β) S5–S6 loop in CP-S by ∼2.5 Å.
(0.40 MB TIF)

Figure S5 **Residues at the intermolecular interface of the CP/CA21 and CP/CK23 complexes.** CP is presented as a surface model with the residues contacting the peptides shown in purple (CP-L domain) and cyan (CP-S domain). The residues of the peptides are underlined. In (A), CA21 is shown as a gray stick model and the conserved residues are highlighted in red. In (B), CK23 is shown as a gray stick model and the conserved residues are highlighted in blue. All of the peptides contact CP residues that reside in the β-subunit, except for (α) Phe168 and Tyr199 (A) and (α) Phe168 (B).
(1.12 MB TIF)

Figure S6 **The importance of the C-terminal flanking region of the CP-binding motif.** (A) Interactions between the C-terminus of CD23 and the CP-L domain. Interface residues are shown as stick models. (B) Bottom view of CP/CARMIL peptide complexes. CP is viewed from the “N-stalk.” CARMIL peptides are shown as tubes. The Cα position of CD Gly507, the C-terminal end residue of CD23, is shown as a green ball. Note that the C-terminus of CD23 points toward the space between the “N-stalk” and “β-globule.” Thus, extension of the peptide at the C-terminus appears to provide additional contacts with CP.
(1.41 MB TIF)

Figure S7 **CD23 and CK23 effectively compete with GST-CA92 for CP binding.** GST-CA92 was immobilized on a sensor chip and then perfused with 50 nM wild type CP pre-mixed with various concentrations of CARMIL peptides. The addition of CK23 effectively inhibited CP from GST-CA92 binding, in a similar manner as CD23. In contrast, CA21 was a less efficient competitor. Note we could not increase the concentration of CA21 higher than 500 nM due to the solubility limit of the peptide.
(0.25 MB TIF)

Figure S8 **CARMIL peptides inhibit CP binding to V-1 in a pull-down assay.** The effect of CARMIL peptides on CP/V-1 complex formation under equilibrium conditions was measured by a pull-down assay. Glutathione sepharose beads were coupled with 2 µM GST-V-1. The beads were incubated for 2 h with 1 µM CP in the presence of various concentrations of CD23 (A), CK23 (B), or CD23 (C). Unbound and bound CP fractions were quantified by SDS-PAGE with CBB staining. G, Glutathione beads were coupled with 2 µM GST and were loaded with CP (no peptides). V, GST-V-1 coupled beads were incubated in the absence of CP and peptides. (D) The amounts of GST-V-1-bound CP in (A–C) were plotted against the concentration of the CARMIL peptides added: CD23 (orange triangle), CK23 (blue square), CA21 (red circle).
(0.56 MB TIF)

Figure S9 **Sequence alignment of V-1.** The amino acid sequences of V-1 proteins from various species were aligned by ClustalW [52]. ANK1-4 denotes the ankyrin repeats. Bars above the sequences represent α-helices or loops. Asterisks mark residues contacting the CP α (yellow) or β (green) subunits. Strictly and highly conserved residues are colored red and yellow, respectively. For ANK2 and 3, the consensus sequence of the ankyrin repeat proteins [53] is aligned below (x, any amino acid except cysteine, glycine, or proline; z, any amino acid except histidine, asparagine, or tyrosine; key residues for the structure are shown in red). A comprehensive sequence analysis of ankyrin repeat proteins demonstrated that these key residues (in red) play crucial roles in maintaining the folding characteristic of the ankyrin repeat protein, a stack of helix-turn-helix bundles, and are well-conserved in most repeats [54],[55]. Note that the CP binding residues including three critical residues, Trp8, Asp44, and Glu78, are strictly conserved among the species, despite not being key residues required for protein folding.
(0.80 MB TIF)

Figure S10 **Fitting analysis of CP to the EM model of the CP/actin filament structure.** The atomic structures of CP in different conformations were fitted to the 3D electron density map of the CP/actin filament complex [5]. The contour level of the EM envelope was set to 130%, and the orientations of CP_full_ (blue) and CP_V-1_ (red) in the model are shown. The viewing angle of CP is shown in the inset. Note that the conformation of CP_full_ provides a better fit to the EM model than CP_V-1_. The cyan arrow indicates a substantial mismatch between the envelope and CP_V-1_. Part of the “β-globule” protrudes out of the envelope, due to the flatter, opened conformation of CP_V-1_ as compared with CP_full_ (see Figures 5A, 5B, and S4), implying that CP may not bind tightly to the barbed end in the “open” conformation.
(0.49 MB TIF)

Figure S11 **Sequence alignment of the CP-binding motif with the C-terminal flanking region of CARMIL proteins.** The amino acid sequence of CARMIL (human, mouse, Dictyostelium, and Acanthamoeba), CD2AP (human), and CKIP-1 (human) are aligned. The CP-binding motif is highlighted in magenta. Basic residues are indicated in blue. Asterisks denote the C-terminus of the protein. During the preparation of this manuscript, Robinson and colleagues reported the crystal structure of CP in complex with a CARMIL fragment which shows full CP inhibition activity (CBR115; human CARMIL residues 964–1078)[36]. This structure revealed a 15 amino acid residue motif that serves as the second CP binding site additional to the CP-binding motif (CARMIL-specific interaction motif, residues 1021–1035; highlighted in orange). Green arrows indicate the C-terminus of CARMIL fragments, GST-CA76, or mCAH3 [29].
(0.33 MB TIF)

Table S1 **Crystallographic statistics.**
(0.05 MB DOC)

Table S2 **Binding affinities between CP and V-1.**
(0.04 MB DOC)

Table S3 **Cα RMSDs between CP crystal structures.**
(0.04 MB DOC)

Table S4 **Small displacement residues in normal mode analysis.**
(0.03 MB DOC)

Text S1 **Supplemental discussion.** The role of the C-terminal flanking region of the CP-binding motif in the CARMIL proteins.
(0.04 MB DOC)

## Abbreviations

CP: capping protein
EM: electron microscopy
ENM: elastic network model
RMSD: root-mean-square displacement
