# Supplementary figures and images for: Two Distinct Mechanisms for Actin Capping Protein Regulation—Steric and Allosteric Inhibition
Source: PLoS Biol. 2010 Jul 6;8(7):e1000416. doi: 10.1371/journal.pbio.1000416 (PMC2897767; doi:10.1371/journal.pbio.1000416)

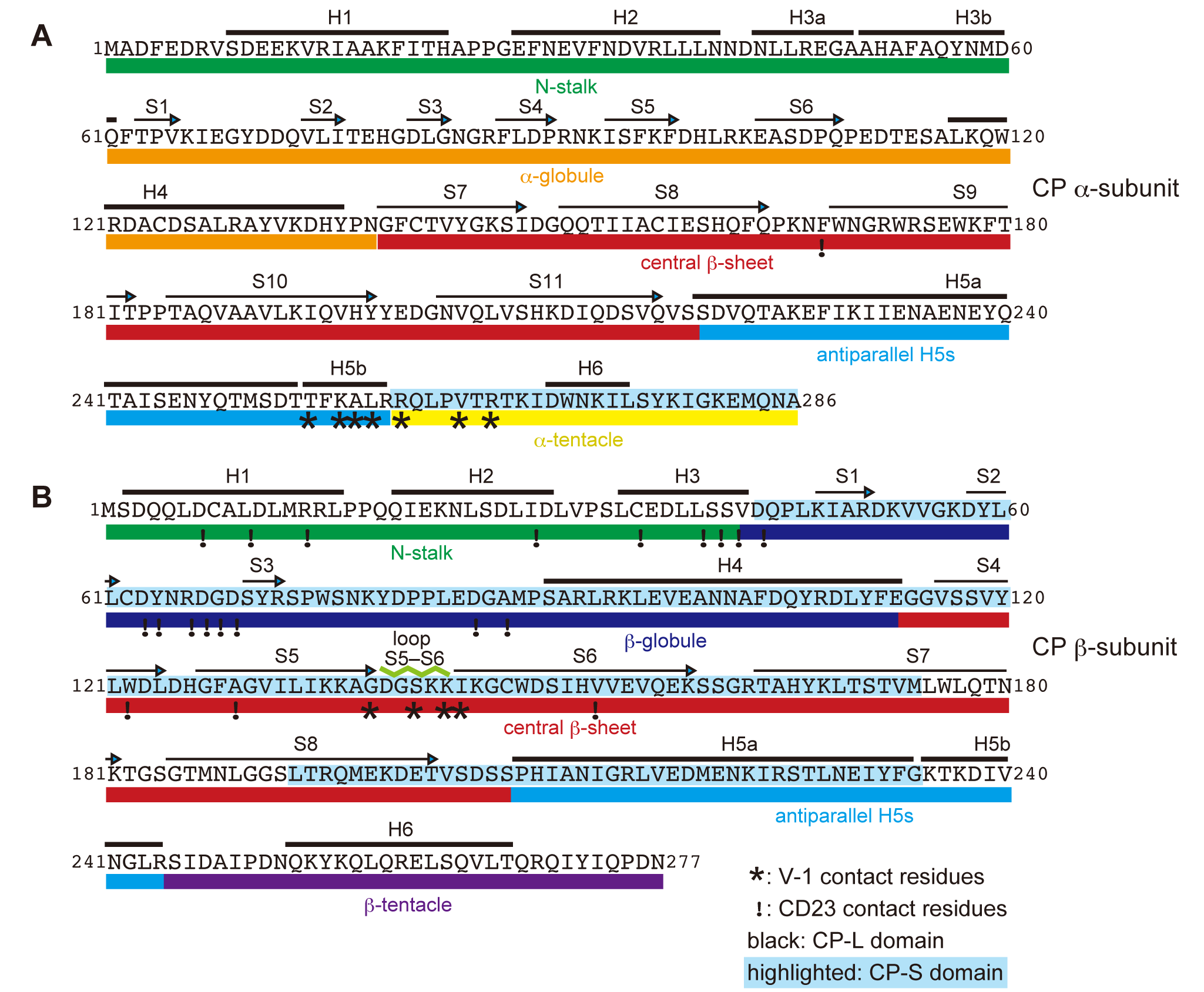

Supplement: Figure S1 — Primary sequence of CP. The amino acid sequences of the chicken CP α1-subunit (A) and β1-subunit (B) are shown. Bars and arrows above the sequences represent α-helices and β-strands, respectively; asterisks and exclamation marks indicate the V-1 and CD23 interacting residues, respectively. The loop S5–S6 (β), which is important for V-1 binding, is indicated as a green wavy line. Residues in the CP-S domain are highlighted in cyan. Each structural motif is indicated with a bar underneath, in the colors corresponding to those in Figure 1A. At the N-terminus, each CP subunit has three α-helices (we call this region the “N-stalk”). On each side of the “N-stalk,” short β-strands are packed in a unique manner to form globular structures that are flanked by helix 4 of either subunit (“α-globule” and “β-globule”). A large anti-parallel β-sheet consisting of 10 β-strands forms the central layer (“central β-sheet”). Helix 5 of both subunits, containing a helix-breaking residue [Thr253 (α) and Gly234 (β)], lies above the “central β-sheet” in an anti-parallel fashion (“antiparallel H5s”), and each is flanked by C-terminal extensions that possess barbed end capping activity (“α-tentacle” and “β-tentacle”). (0.39 MB TIF) [file pbio.1000416.s001.tif]

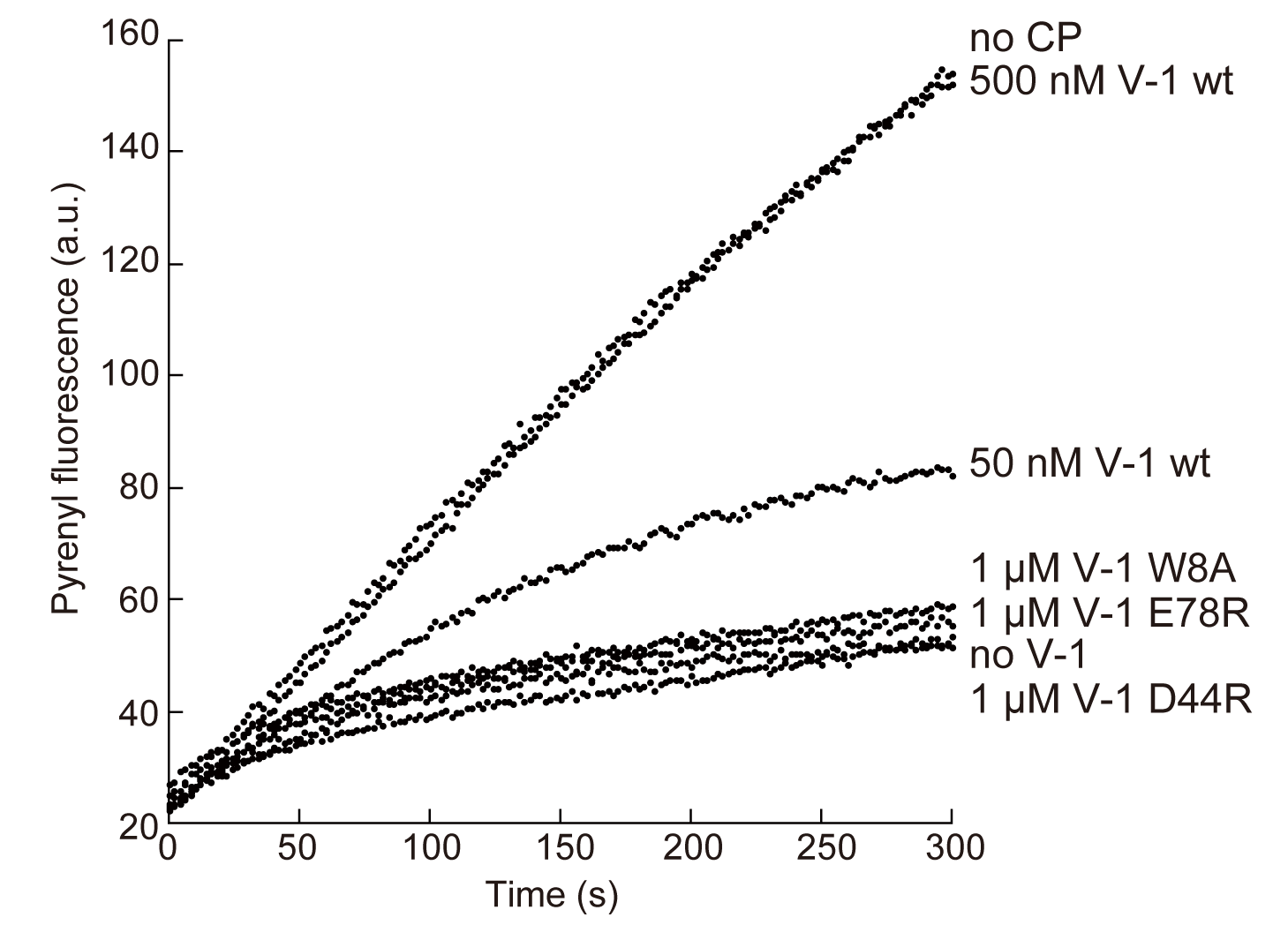

Supplement: Figure S2 — V-1 mutants deficient in CP-binding fail to inhibit CP's capping activity. Actin (1.2 µM; 5% pyrene-labeled) was polymerized from spectrin-actin seeds in the presence of 3 nM wild type CP with various concentrations of wild type V-1 or mutant V-1 proteins. The addition of wild-type V-1 to the system at a concentration well above the KD inhibited CP from barbed end capping. In contrast, the CP-binding deficient V-1 mutants (V-1 W8A, D44R, or E78R; see Table S2) showed minimal effects on CP capping. (0.16 MB TIF) [file pbio.1000416.s002.tif]

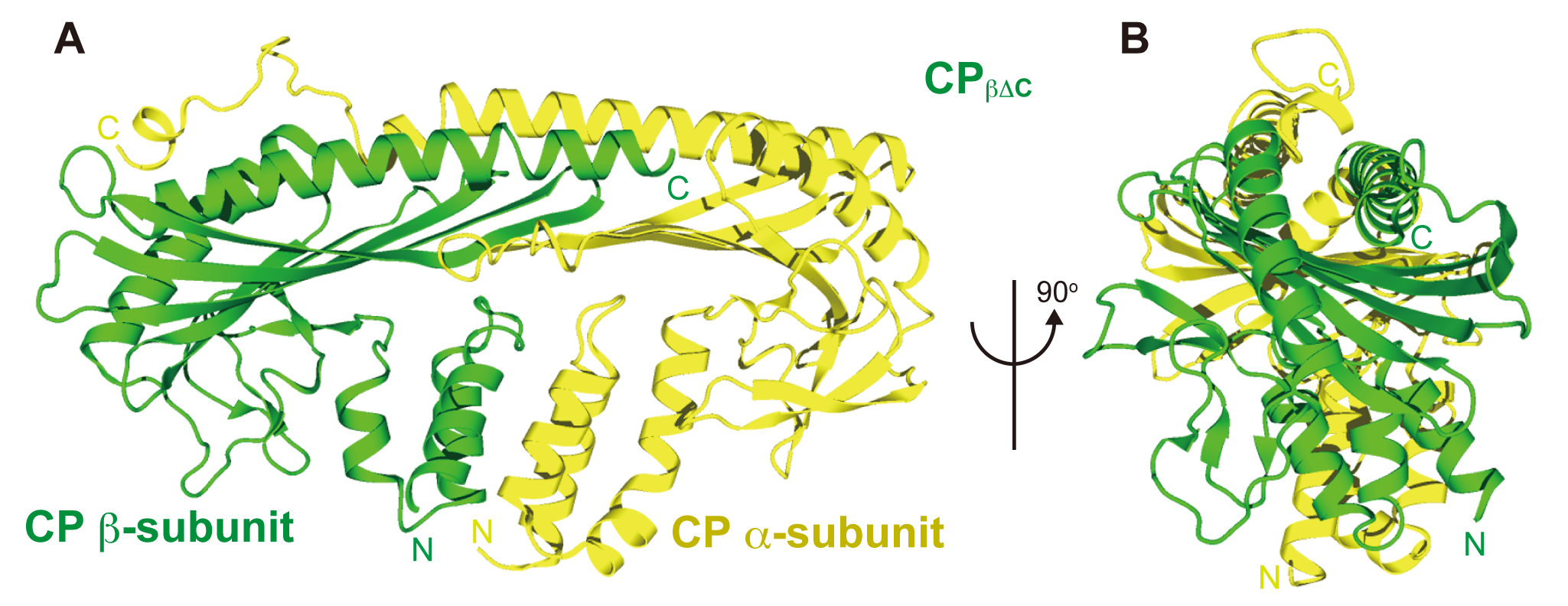

Supplement: Figure S3 — Crystal structure of CPβΔC. Crystal structure of CPβΔC (chicken α1 full/β1 1–244; a deletion mutant CP lacking the “β-tentacle”) at 1.9 Å resolution. Two orthogonal views of the structure are shown in ribbon models with the α-subunit (yellow) and β-subunit (green). The N- and C-termini are indicated. The secondary structures of CPβΔC are nearly identical to those of CPfull, although the overall conformations are significantly different between the two structures (see Table S3). (1.04 MB TIF) [file pbio.1000416.s003.tif]

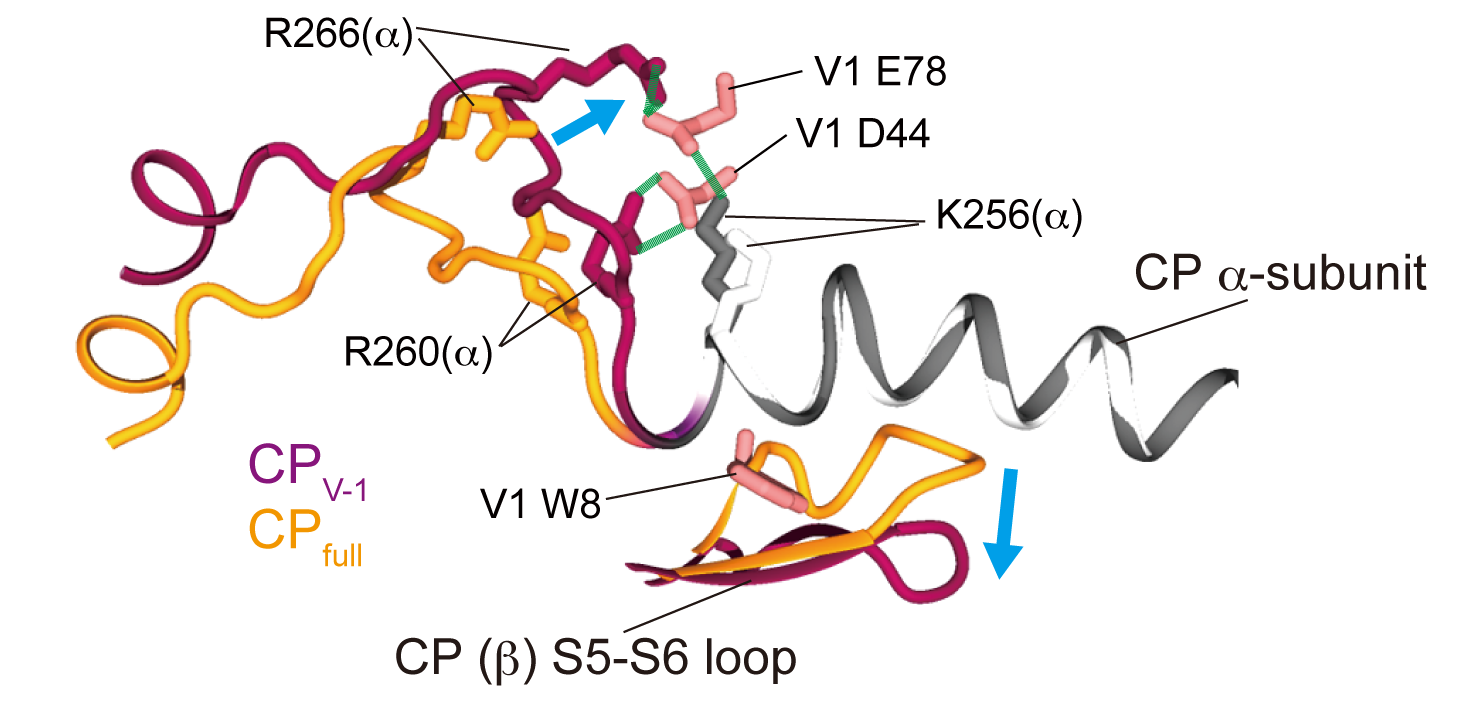

Supplement: Figure S4 — Structural change to CP induced by V-1 binding. The C-terminal region of the CP α-subunit (residues 243–275) and the loop (β) S5–S6 are shown in ribbon models. CPfull: CP-L (white) and CP-S (orange): CPV-1: CP-L (gray) and CP-S (purple); V-1: magenta. Molecular interface residues [CP (α) Lys256, Arg260 and Arg266, V-1 Trp8, Asp44 and Glu78] are shown as stick models and hydrogen bonds are indicated by green lines. Shifts of CPV-1 induced by the V-1 interaction are represented by cyan arrows. Upon V-1 binding, the CP-S domain rotates by approximately 10° relative to the CP-L domain about a rotation axis that is nearly identical to the long axis of the molecule (see Figure 5F and 5G). Since V-1 Glu78 interacts simultaneously with CP (α) Lys256 near the domain boundary and with Arg266 in the “α-tentacle,” it pulls Arg266 towards Lys256 by ∼2.7 Å (Cα positions). This shift straightens the “antiparallel H5s” and further moves the rest of CP-S bound tightly with the “α-tentacle.” As a result, the distance between the “N-stalk” and “β-globule” becomes wider (see Figure 5A and 5B). Simultaneously, V-1 ANK 1 pushes down the CP (β) S5–S6 loop in CP-S by ∼2.5 Å. (0.40 MB TIF) [file pbio.1000416.s004.tif]

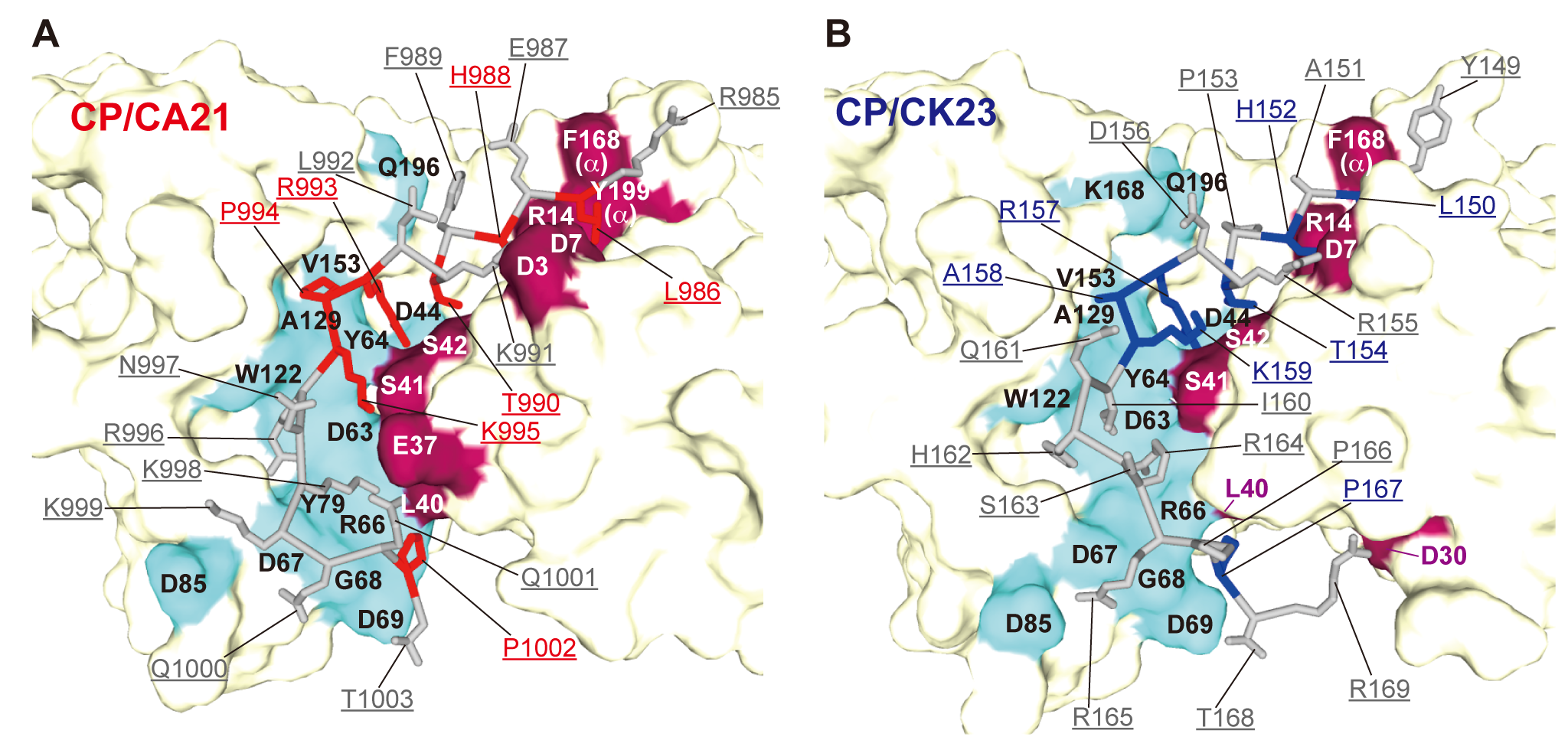

Supplement: Figure S5 — Residues at the intermolecular interface of the CP/CA21 and CP/CK23 complexes. CP is presented as a surface model with the residues contacting the peptides shown in purple (CP-L domain) and cyan (CP-S domain). The residues of the peptides are underlined. In (A), CA21 is shown as a gray stick model and the conserved residues are highlighted in red. In (B), CK23 is shown as a gray stick model and the conserved residues are highlighted in blue. All of the peptides contact CP residues that reside in the β-subunit, except for (α) Phe168 and Tyr199 (A) and (α) Phe168 (B). (1.12 MB TIF) [file pbio.1000416.s005.tif]

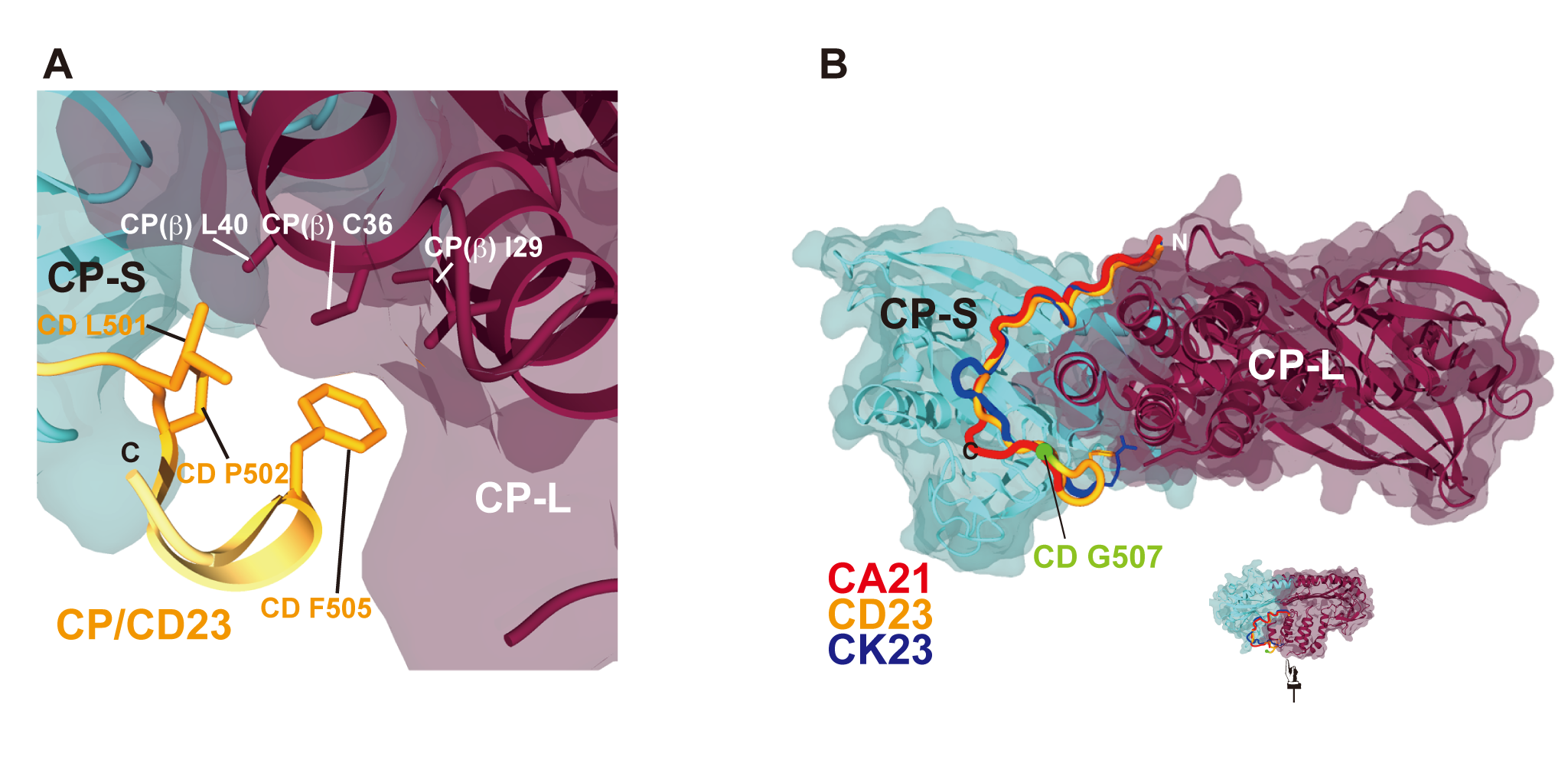

Supplement: Figure S6 — The importance of the C-terminal flanking region of the CP-binding motif. (A) Interactions between the C-terminus of CD23 and the CP-L domain. Interface residues are shown as stick models. (B) Bottom view of CP/CARMIL peptide complexes. CP is viewed from the “N-stalk.” CARMIL peptides are shown as tubes. The Cα position of CD Gly507, the C-terminal end residue of CD23, is shown as a green ball. Note that the C-terminus of CD23 points toward the space between the “N-stalk” and “β-globule.” Thus, extension of the peptide at the C-terminus appears to provide additional contacts with CP. (1.41 MB TIF) [file pbio.1000416.s006.tif]

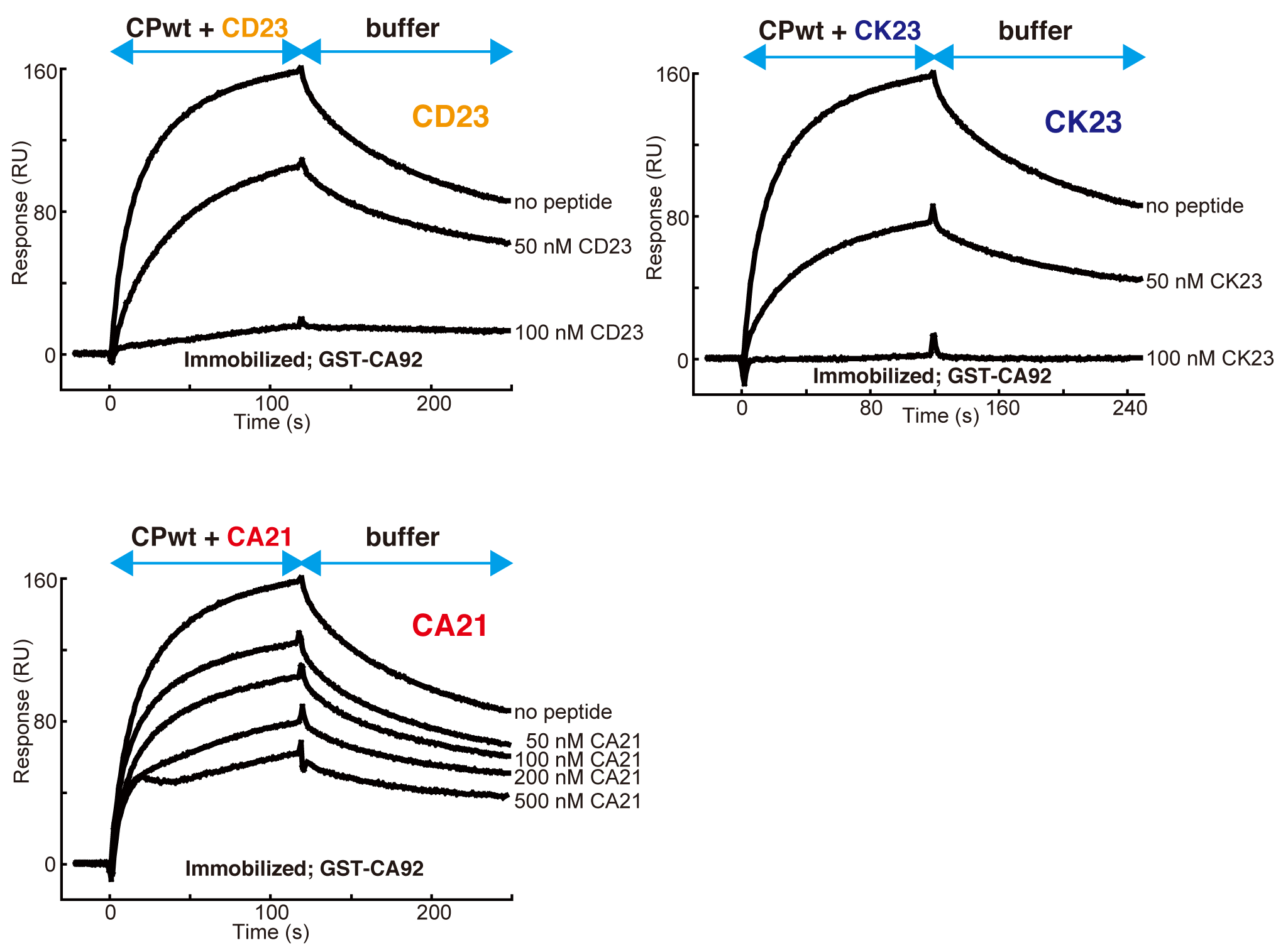

Supplement: Figure S7 — CD23 and CK23 effectively compete with GST-CA92 for CP binding. GST-CA92 was immobilized on a sensor chip and then perfused with 50 nM wild type CP pre-mixed with various concentrations of CARMIL peptides. The addition of CK23 effectively inhibited CP from GST-CA92 binding, in a similar manner as CD23. In contrast, CA21 was a less efficient competitor. Note we could not increase the concentration of CA21 higher than 500 nM due to the solubility limit of the peptide. (0.25 MB TIF) [file pbio.1000416.s007.tif]

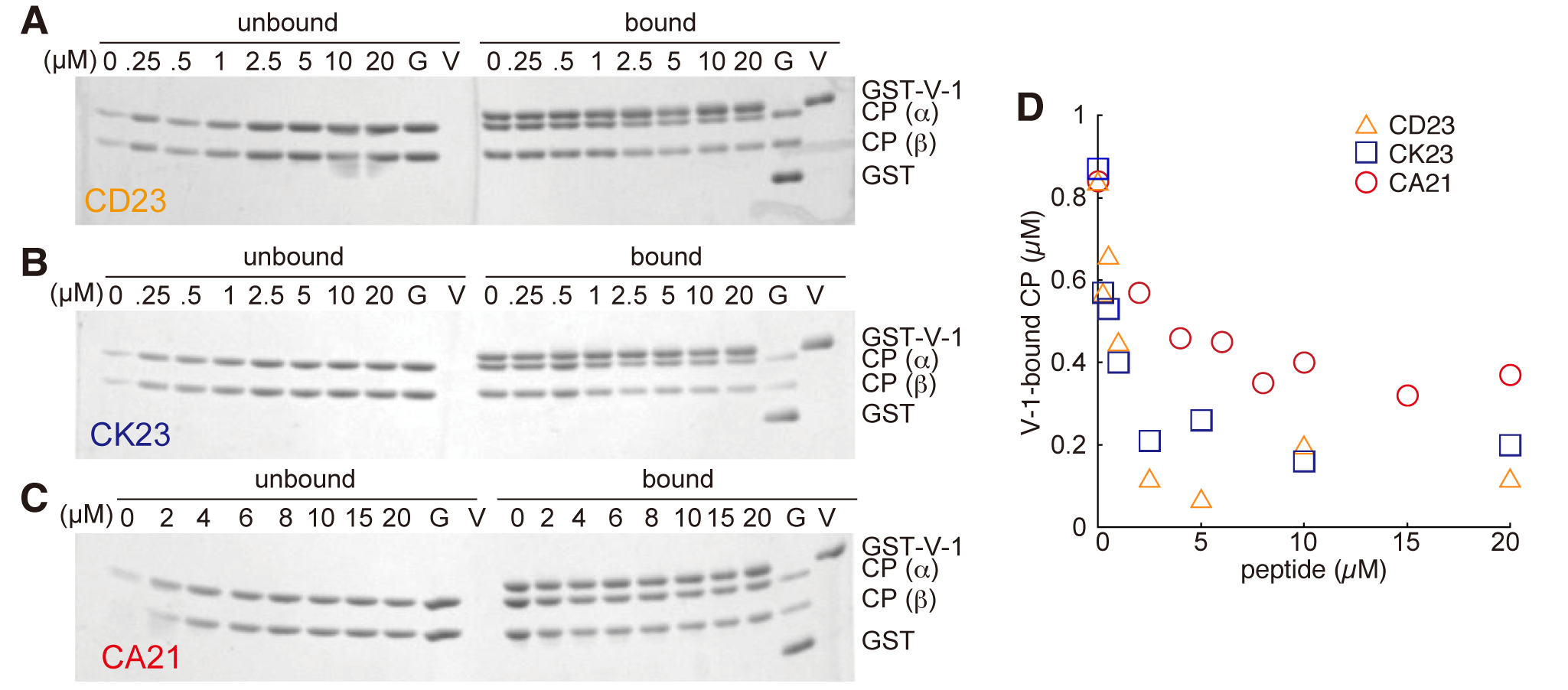

Supplement: Figure S8 — CARMIL peptides inhibit CP binding to V-1 in a pull-down assay. The effect of CARMIL peptides on CP/V-1 complex formation under equilibrium conditions was measured by a pull-down assay. Glutathione sepharose beads were coupled with 2 µM GST-V-1. The beads were incubated for 2 h with 1 µM CP in the presence of various concentrations of CD23 (A), CK23 (B), or CD23 (C). Unbound and bound CP fractions were quantified by SDS-PAGE with CBB staining. G, Glutathione beads were coupled with 2 µM GST and were loaded with CP (no peptides). V, GST-V-1 coupled beads were incubated in the absence of CP and peptides. (D) The amounts of GST-V-1-bound CP in (A–C) were plotted against the concentration of the CARMIL peptides added: CD23 (orange triangle), CK23 (blue square), CA21 (red circle). (0.56 MB TIF) [file pbio.1000416.s008.tif]

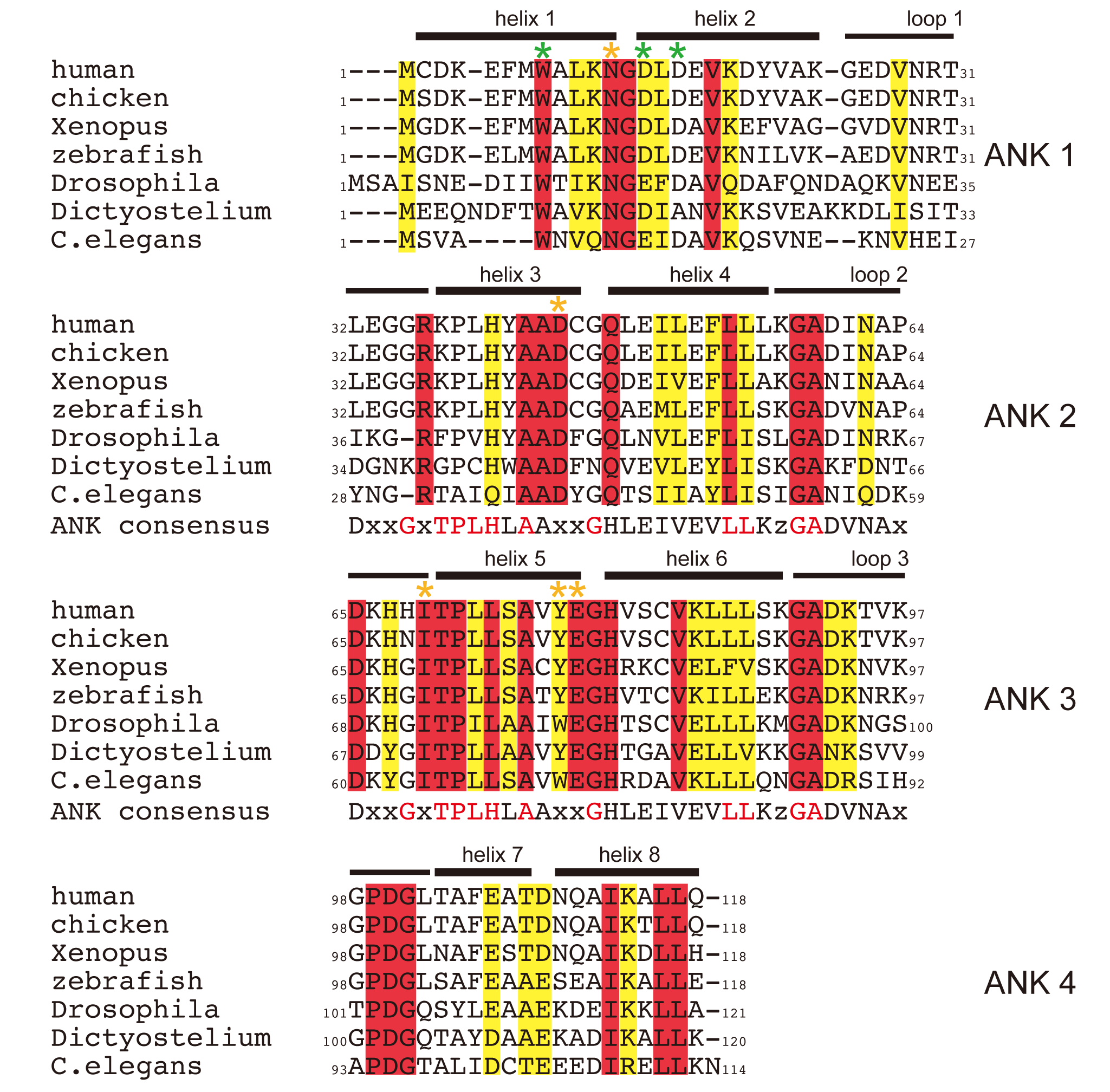

Supplement: Figure S9 — Sequence alignment of V-1. The amino acid sequences of V-1 proteins from various species were aligned by ClustalW [52]. ANK1-4 denotes the ankyrin repeats. Bars above the sequences represent α-helices or loops. Asterisks mark residues contacting the CP α (yellow) or β (green) subunits. Strictly and highly conserved residues are colored red and yellow, respectively. For ANK2 and 3, the consensus sequence of the ankyrin repeat proteins [53] is aligned below (x, any amino acid except cysteine, glycine, or proline; z, any amino acid except histidine, asparagine, or tyrosine; key residues for the structure are shown in red). A comprehensive sequence analysis of ankyrin repeat proteins demonstrated that these key residues (in red) play crucial roles in maintaining the folding characteristic of the ankyrin repeat protein, a stack of helix-turn-helix bundles, and are well-conserved in most repeats [54],[55]. Note that the CP binding residues including three critical residues, Trp8, Asp44, and Glu78, are strictly conserved among the species, despite not being key residues required for protein folding. (0.80 MB TIF) [file pbio.1000416.s009.tif]

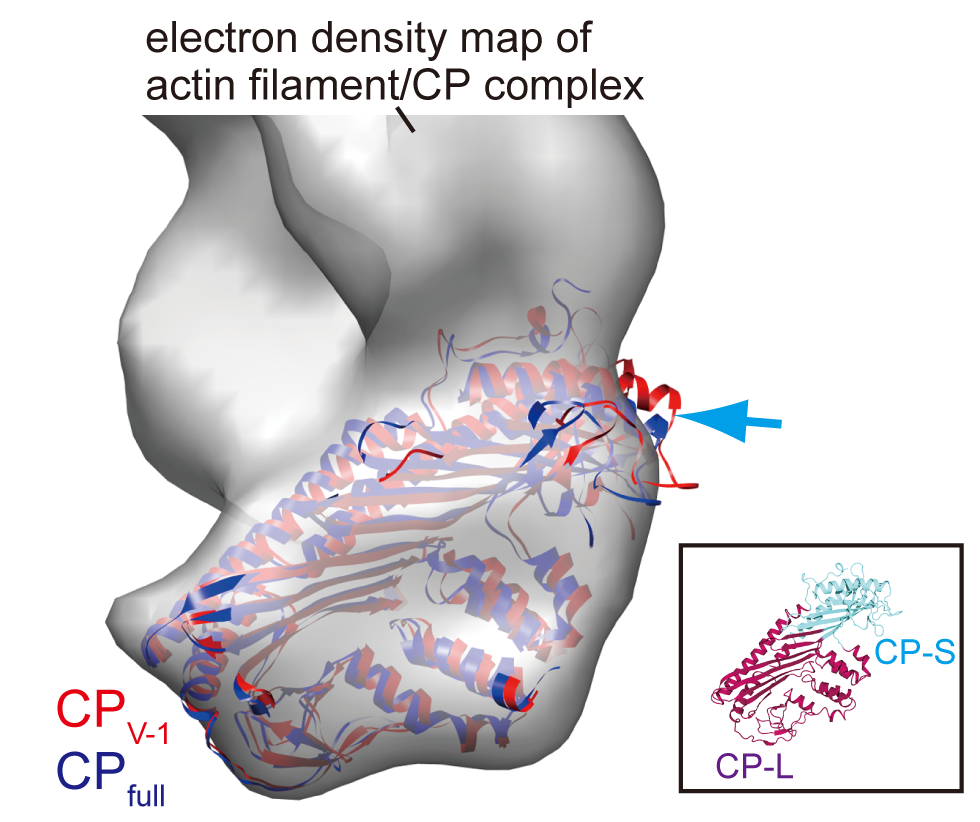

Supplement: Figure S10 — Fitting analysis of CP to the EM model of the CP/actin filament structure. The atomic structures of CP in different conformations were fitted to the 3D electron density map of the CP/actin filament complex [5]. The contour level of the EM envelope was set to 130%, and the orientations of CPfull (blue) and CPV-1 (red) in the model are shown. The viewing angle of CP is shown in the inset. Note that the conformation of CPfull provides a better fit to the EM model than CPV-1. The cyan arrow indicates a substantial mismatch between the envelope and CPV-1. Part of the “β-globule” protrudes out of the envelope, due to the flatter, opened conformation of CPV-1 as compared with CPfull (see Figures 5A, 5B, and S4), implying that CP may not bind tightly to the barbed end in the “open” conformation. (0.49 MB TIF) [file pbio.1000416.s010.tif]

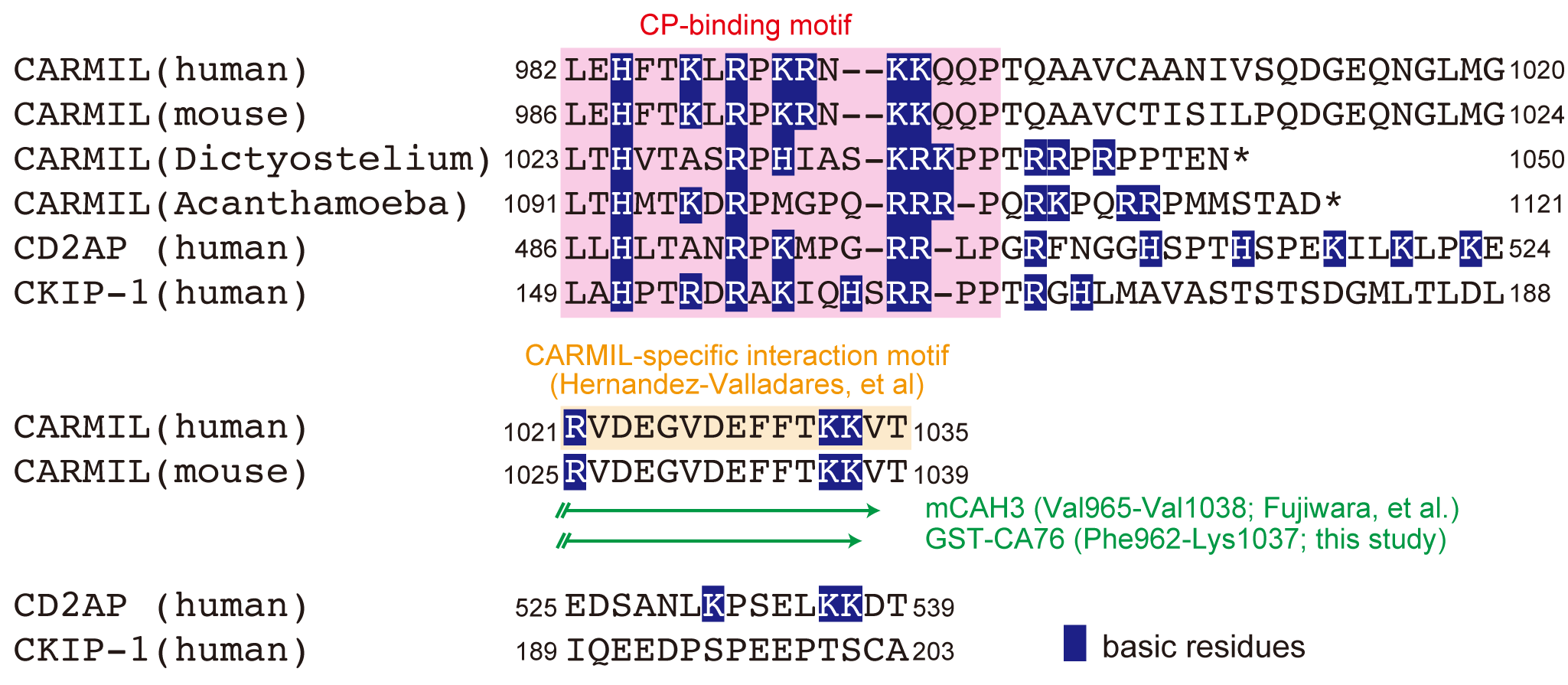

Supplement: Figure S11 — Sequence alignment of the CP-binding motif with the C-terminal flanking region of CARMIL proteins. The amino acid sequence of CARMIL (human, mouse, Dictyostelium, and Acanthamoeba), CD2AP (human), and CKIP-1 (human) are aligned. The CP-binding motif is highlighted in magenta. Basic residues are indicated in blue. Asterisks denote the C-terminus of the protein. During the preparation of this manuscript, Robinson and colleagues reported the crystal structure of CP in complex with a CARMIL fragment which shows full CP inhibition activity (CBR115; human CARMIL residues 964–1078)[36]. This structure revealed a 15 amino acid residue motif that serves as the second CP binding site additional to the CP-binding motif (CARMIL-specific interaction motif, residues 1021–1035; highlighted in orange). Green arrows indicate the C-terminus of CARMIL fragments, GST-CA76, or mCAH3 [29]. (0.33 MB TIF) [file pbio.1000416.s011.tif]
