# Supplementary material for: Two Distinct Mechanisms for Actin Capping Protein Regulation—Steric and Allosteric Inhibition
Source: PLoS Biol. 2010 Jul 6;8(7):e1000416. doi: 10.1371/journal.pbio.1000416 (PMC2897767; doi:10.1371/journal.pbio.1000416)
Supplement: Table S1 — Crystallographic statistics. (0.05 MB DOC) [file pbio.1000416.s012.doc]

**Table S1. Crystallographic Statistics.**

| Analyzed Structure Determination Step | Static Type | CP/V-1 | CP_βΔC_ | CP/CA21 | CP/CD23 | CP/CK23 |
| --- | --- | --- | --- | --- | --- | --- |
| Data collection | Resolution (Å) | 20.00-2.20 (2.28-2.20) | 50.0-1.90 (1.97-1.90) | 50.0-1.7 (1.76-1.70) | 50.0-1.90 (1.97-1.90) | 50.0-1.90 (1.97-1.90) |
|  | Space group | P2_1_2_1_2_1_ | P2_1_2_1_2_1_ | P2_1_2_1_2_1_ | P2_1_2_1_2_1_ | P2_1_2_1_2_1_ |
|  | Cell dimensions (a/b/c; Å) | 71.42/87.01/121.0 | 53.91/68.68/130.1 | 54.81/67.92/137.1 | 59.12/63.87/141.1 | 57.32/66.30/136.7 |
|  | Unique reflections | 38745 (3818) | 37190 (2944) | 55697 (4405) | 41167 (3026) | 40661 (3337) |
|  | Redundancy | 14.1 (13.8) | 6.4 (4.6) | 6.1 (2.5) | 6.6 (4.6) | 6.5 (4.0) |
|  | I/σ(I) | 24.62 (7.68) | 15.56 (4.92) | 18.32 (2.03) | 17.43 (4.71) | 17.66 (4.41) |
|  | R_merge_ (I) | 0.102 (0.304) | 0.068 (0.290) | 0.080 (0.348) | 0.062 (0.282) | 0.059 (0.261) |
|  | Completeness (%) | 100.0 (100.0) | 96.2 (77.7) | 97.7 (78.7) | 96.0 (71.8) | 97.4 (81.7) |
| Refinement | Resolution (Å) | 19.96-2.20 | 49.80-1.90 | 42.81-1.70 | 45.31-1.90 | 43.36-1.90 |
|  | R_work_/R_free_ | 0.186/0.237 | 0.209/0.261 | 0.192/0.239 | 0.184/0.238 | 0.213/0.263 |
|  | Rmsd bond length (Å) | 0.015 | 0.015 | 0.015 | 0.015 | 0.015 |
|  | Rmsd bond angles (º) | 1.42 | 1.53 | 1.56 | 1.51 | 1.53 |

Values in parentheses refer to the highest resolution shell.
