# Supplementary material for: Two Distinct Mechanisms for Actin Capping Protein Regulation—Steric and Allosteric Inhibition
Source: PLoS Biol. 2010 Jul 6;8(7):e1000416. doi: 10.1371/journal.pbio.1000416 (PMC2897767; doi:10.1371/journal.pbio.1000416)
Supplement: Table S2 — Binding affinities between CP and V-1. (0.04 MB DOC) [file pbio.1000416.s013.doc]

**Table S2.** **Binding affinities between CP and V-1.**

| immobilized / analyte | k_on_ (µM^-1^s^-1^) | k_off_ (s^-1^) | K_D_ (µM) |
| --- | --- | --- | --- |
| V-1 wt / CP_full_ | 1.5 | 0.031 | 0.021 ^a^ |
| V-1 wt / CP_βΔC_ | 1.8 | 0.038 | 0.022 ^a^ |
| V-1 wt / CP (α) K256A |  |  | 1.4 ^b^ |
| V-1 wt / CP (α) K256E |  |  | 7.1 ^b^ |
| V-1 wt / CP (α) R260A |  |  | 3.6 ^b^ |
| V-1 wt / CP (α) R260E |  |  | 18 ^b^ |
| V-1 wt / CP (α) R266A |  |  | 0.93 ^b^ |
| V-1 wt / CP (α) R266E |  |  | 8.6 ^b^ |
| V-1 wt / CP (α) KR256 266AA |  |  | 69 ^b^ |
| V-1 wt / CP (α) KR256 266EE |  |  | not detectable ^c^ |
| V-1 wt / CP (α) RR256 266AA or EE |  |  | not detectable ^c^ |
| V-1 wt / CP (α) RR260 266AA or EE |  |  | not detectable ^c^ |
| V-1 W8A / CP wt |  |  | 6.4 ^b^ |
| V-1 D44A / CP wt |  |  | 120 ^b^ |
| V-1 D44R / CP wt |  |  | not detectable ^c^ |
| V-1 E78A / CP wt |  |  | 0.58 ^b^ |
| V-1 E78R / CP wt |  |  | 21 ^b^ |

^a^ Kinetic parameters were determined by fitting of the sensorgrams to a 1:1 binding model. K_D_ = k_off_ / k_on_ .

^b^ Kinetic parameters were not determined for mutant proteins, due to the fast dissociation of the complex (k_off_ > 0.1 s^-1^). Instead, equilibrium K_D_ values were obtained from Michaelis-Menten plots at the saturated binding conditions.

^c^ Bindings were not observed even at the highest concentrations tested (≥ 50 µM).
