# Supplementary material for: Two Distinct Mechanisms for Actin Capping Protein Regulation—Steric and Allosteric Inhibition
Source: PLoS Biol. 2010 Jul 6;8(7):e1000416. doi: 10.1371/journal.pbio.1000416 (PMC2897767; doi:10.1371/journal.pbio.1000416)
Supplement: Table S3 — Cα RMSDs between CP crystal structures. (0.04 MB DOC) [file pbio.1000416.s014.doc]

**Table S3. Cα RMSDs between CP crystal structures.**

|  | CP_β∆C_ | CP_CA21_ | CP_CD23_ | CP_CK23_ | CP_full_ |
| --- | --- | --- | --- | --- | --- |
| CP_V-1_ | 1.87 / 0.80 / 1.04 | 2.20 / 0.87 / 1.18 | 2.29 / 0.82 /1.09 | 2.50 / 1.04 / 1.13 | 2.55 / 1.06 / 0.99 |
| CP_β∆C_ | - | 0.97 / 0.61 / 0.57 | 1.04 / 0.55 / 0.74 | 1.26 / 0.78 / 0.71 | 1.34 / 1.04 / 0.80 |
| CP_CA21_ | - | - | 0.71 / 0.56 / 0.84 | 0.86 / 0.91 / 0.54 | 1.07 / 0.95 / 0.94 |
| CP_CD23_ | - | - | - | 0.90 / 0.80 / 0.94 | 1.02 / 1.01 / 0.88 |
| CP_CK23_ | - | - | - | - | 1.07 / 1.00 / 0.97 |

Overall {residues 9–275 (α) and 3–244 (β); β-tentacle was not included} / CP-L domain / CP-S domain (Å)
