# Supplementary material for: Two Distinct Mechanisms for Actin Capping Protein Regulation—Steric and Allosteric Inhibition
Source: PLoS Biol. 2010 Jul 6;8(7):e1000416. doi: 10.1371/journal.pbio.1000416 (PMC2897767; doi:10.1371/journal.pbio.1000416)
Supplement: Table S4 — Small displacement residues in normal mode analysis. (0.03 MB DOC) [file pbio.1000416.s015.doc]

**Table S4**. Small displacements residues in normal mode analysis.

|  | rotational axis through α-subunit | rotational axis through β-subunit |
| --- | --- | --- |
| free CP | 62, 74, 75, 88, 89, 94, 95, 109, 110, 158, 159, 172-175, 192-194, 210, 238-242 (α), 182, 184 (β) | 101, 102, 104, 132, 150, 151, 172, 173, 193, 194 (β) |
| CP/CD23 | 90, 93, 142, 157-159, 173-175, 192-194, 238-243 (α), 182, 184, 241 (β) | 39 (α), 5-14, 32, 42-44, 99-104, 119, 131-133, 151-153, 171, 172, 194 (β), 486-491 (CD23) |
