# Supplementary material for: Two Distinct Mechanisms for Actin Capping Protein Regulation—Steric and Allosteric Inhibition
Source: PLoS Biol. 2010 Jul 6;8(7):e1000416. doi: 10.1371/journal.pbio.1000416 (PMC2897767; doi:10.1371/journal.pbio.1000416)
Supplement: Text S1 — Supplemental discussion. The role of the C-terminal flanking region of the CP-binding motif in the CARMIL proteins. (0.04 MB DOC) [file pbio.1000416.s016.doc]

**Text S1. Supplemental discussion. The role of the C-terminal flanking region of the CP-binding motif in the CARMIL proteins**

Uruno et al. reported that, in contrast to mammalian CARMILs, Acanthamoeba CARMIL, which has only ~15 residues C-terminal to the CP-binding motif {51 aa (residues 1071−1121)}, effectively inhibits CP activity ([1] and Figure S11). The CP surface is highly negatively charged (calculated pI is 5.6) except for the “basic triad” region on the “α-tentacle” which constitutes a binding site for the barbed end and V-1, thus basic residues in the CP-binding proteins would be expected to play an important role for CP interaction. Several lines of evidence support this notion. (1) The CP-binding motif of CARMIL proteins is highly basic (Figure 6A). (2) In CP/CK23 complex, CK Arg169 is involved in the interaction with CP-L domain residues (Figure S5B) and in CD2AP, CD Arg504 provides CP binding stability (see the difference in the K_D_ between GST-CD43 and CD44; Table 2). (3) In contrast to CD2AP and CKIP-1, mammalian CARMILs lack basic residues in the region immediately C-terminal to the conserved motif (Figure S11). Mammalian CARMILs that have the conserved motif with the short C-terminal flanking regions (i.e., GST-CA55, 63 and CA21) bind CP only weakly (Table 2 and Figure S7). (4) A recently reported crystal structure of CP in complex with a longer CARMIL fragment (CBR115: human residues 964−1078) [2] revealed a second CP binding motif (the CARMIL-specific interaction motif: residues 1021−1035) located C-terminal to the CP-binding motif. The second CP-binding motif starts with human CARMIL Arg1021 which is the first basic residue C-terminal to the conserved motif (Figure S11).

Based on these findings, we assume that the 51 aa amoeba CARMIL fragment is highly active because it posses several basic residue in the C-terminal flanking region of the conserved motif (Figure S11). Thus, amoeba CARMIL may inhibit CP in a manner similar to CD2AP or CKIP-1.

Fujiwara, et al. reported that the mCAH3 fragment (mouse CARMIL residues 965−1038) tightly binds to CP (KD ~ 1 nM) whereas GST-CA76 (residues 962−1037) in the present study interacts weakly with CP (Table 2). In the second CP binding site in the CP/CBR115 complex [2], human CARMIL Val1034 (corresponding to mouse CA1038), which is missing in GST-76, forms multiple interactions with CP α-subunit residues. Thus, the absence of this key valine in GST-CA76 results in the lower CP binding affinity compared with the full active CARMIL fragments.

**References for Text S1.**

1. Uruno T, Remmert K, Hammer JA, 3rd (2006) CARMIL is a potent capping protein antagonist: identification of a conserved CARMIL domain that inhibits the activity of capping protein and uncaps capped actin filaments. J Biol Chem 281: 10635-10650.

2. Hernandez-Valladares M, Kim T, Kannan B, Tung A, Aguda AH, et al. (2010) Structural characterization of a capping protein interaction motif defines a family of actin filament regulators. Nat Struct Mol Biol 17: 497-503.

3. Fujiwara I, Remmert K, Hammer JA (2009) Direct observation of the uncapping of capping protein-capped actin filaments by CARMIL homology domain 3 (CAH3). J Biol Chem.
